# Quantitative assessment of the susceptibility artefact and its interaction with motion in diffusion MRI

**DOI:** 10.1371/journal.pone.0185647

**Published:** 2017-10-02

**Authors:** Mark S. Graham, Ivana Drobnjak, Mark Jenkinson, Hui Zhang

**Affiliations:** 1 Centre for Medical Image Computing & Department of Computer Science, University College London, London, United Kingdom; 2 Wellcome Centre for Integrative Neuroimaging, FMRIB, Nuffield Department of Clinical Neurosciences, University of Oxford, Oxford, United Kingdom; University of North Carolina at Chapel Hill, UNITED STATES

## Abstract

In this paper we evaluate the three main methods for correcting the susceptibility-induced artefact in diffusion-weighted magnetic-resonance (DW-MR) data, and assess how correction is affected by the susceptibility field’s interaction with motion. The susceptibility artefact adversely impacts analysis performed on the data and is typically corrected in post-processing. Correction strategies involve either registration to a structural image, the application of an acquired field-map or the use of additional images acquired with different phase-encoding. Unfortunately, the choice of which method to use is made difficult by the absence of any systematic comparisons of them. In this work we quantitatively evaluate these methods, by extending and employing a recently proposed framework that allows for the simulation of realistic DW-MR datasets with artefacts. Our analysis separately evaluates the ability for methods to correct for geometric distortions and to recover lost information in regions of signal compression. In terms of geometric distortions, we find that registration-based methods offer the poorest correction. Field-mapping techniques are better, but are influenced by noise and partial volume effects, whilst multiple phase-encode methods performed best. We use our simulations to validate a popular surrogate metric of correction quality, the comparison of corrected data acquired with AP and LR phase-encoding, and apply this surrogate to real datasets. Furthermore, we demonstrate that failing to account for the interaction of the susceptibility field with head movement leads to increased errors when analysing DW-MR data. None of the commonly used post-processing methods account for this interaction, and we suggest this may be a valuable area for future methods development.

## Introduction

Diffusion-weighted magnetic resonance (DW-MR) imaging is a powerful technique that enables us to non-invasively probe tissue microstructure. It has proved an invaluable tool for the study of white matter [1] and, more recently, is being applied to the study of grey matter [2, 3]. Recent advances in acquisition—such as simultaneous multi-slice (SMS) imaging—are providing diffusion datasets of unprecedented quality [4], enabling the increasingly rich characterisation of tissue microstructure.

Unfortunately analysis of DW-MR data is confounded by the presence of the susceptibility artefact, caused by an off-resonance field induced by differences in magnetic susceptibility at the air-tissue interface. When images are acquired with the spin-echo (SE) echo-planar imaging (EPI) sequence typically used in DW-MRI [5] this field causes geometric distortions in the data. If the subject remains static during acquisition these geometric distortions will be the same for each volume, resulting in diffusion datasets that are internally consistent (every volume contains the same distortions) but not anatomically faithful (the volumes do not match the subject’s true anatomy). This has been shown to preclude accurate alignment to anatomically faithful structural data [6], a step that is often necessary for localising fine structures in the diffusion data, and to also introduce bias into results obtained from tractography [7–9]. We refer to this situation as the static susceptibility case. If the subject moves during acquisition the susceptibility field itself changes [10], altering the geometric distortions in the data, meaning that even after rigid realignment to correct for motion the diffusion datasets are both geometrically distorted and internally inconsistent (DW-MR volumes are misaligned relative to each other). In this case even analysis of the data that is not dependent on anatomical faithfulness, such as voxelwise fits to the data, will suffer from increased variability. We refer to this movement-induced change to the susceptibility field as the dynamic portion of the susceptibility artefact.

Recent trends in DW-MR are making it increasingly important that we have robust, well validated techniques for correcting this artefact. In the recent past, it has been common to reduce the impact of the susceptibility artefact at scan-time, by using in-plane parallel imaging techniques to reduce the number of phase-encoding (PE) steps. However, recently a number of high-profile studies such as the Human Connectome Project (HCP), HCP lifespan and the UK Biobank have chosen to forego the use of these techniques in favour of SMS methods [11, 12], citing instabilities in reconstruction when the two are employed together [13, 14]. As these acquisition choices filter down to more ‘everyday’ studies there will be a concurrent increase in the severity of the susceptibility artefact. Furthermore, there is a trend, partly facilitated by the ability to image faster, towards acquiring datasets in more ‘difficult’ populations such as in the developing HCP [15]. These populations tend to move more in the scanner, further exacerbating problems caused by the interaction between susceptibility and motion.

There are a number of techniques available for correcting the susceptibility artefact. Correction is usually undertaken using post-processing strategies that may require the collection of some additional data. Broadly, these techniques can be divided into three types. The first involves registration of the data to a geometrically correct structural image [16–23]. The second type estimates a map of the *B*_0_ inhomogeneities from acquired gradient-echo scans, and uses this along with some information about the diffusion acquisition protocol to correct for the distortions [24–28]. The third estimates the underlying distortions using additional EPI data that is acquired with different phase-encoding (PE) and thus contains different distortions [29–35]. This last class of techniques offer the additional opportunity to accurately recover lost signal information if the full dataset has been acquired with reversed phase-encoding.

There are two classes of correction technique that rely on specialised pulse sequences not currently available on most scanners. Multi-reference approaches [36–38] are similar to fieldmap-based methods in that they involve the acquisition of additional reference scans to measure the geometric distortions in the data and correct in post-processing. Scan-time correction schemes estimate the fieldmap in real-time and correct it using gradient shims [39, 40]. These techniques are less commonly used and not examined in this paper.

The majority of post-processing techniques assume a single susceptibility-induced field for the diffusion data and it is difficult to assess the impact of this assumption on the analysis of diffusion data. The problem has been investigated in the context of fMRI [6, 24], and a number of post-processing techniques suggested for its correction [41, 42] but they cannot be used to correct DW-MR data. This is because these methods either assume the off-resonance field can be measured from the phase [42], which is not true for diffusion data where the weighting can alter the phase, or because they assume all undistorted images in a time-series should have the same shape [41], which is not true if the images have different diffusion-weighting, which can alter the apparent location of the brain’s outer surface.

It is important that we have available careful comparisons of susceptibility correction strategies, so that we are able to select the best for our processing pipelines. It is also vital that we are aware of the impact that their inability to correct for the dynamic portion of the artefact has on data analysis. To date, there are no systematic comparisons of existing methods for susceptibility correction and their limitations. A key reason for this is the difficulty in evaluating correction techniques. When validating on real data, the lack of any ground truth means evaluations are typically indirect [18, 34, 43] or qualitative [24, 31, 32]. Furthermore evaluations are often confounded by features in the data that are not of interest, such as other artefacts. Simulation can provide a ground-truth that enables direct, quantitative evaluation, and further allows for the careful design of experiments that enable the direct testing of the artefact of interest, without confounds.

In this work, we use simulation to undertake a comparison of the three classes of technique used for correction of the susceptibility artefact, and further characterise the impact of their inability to correct for the dynamic portion of the artefact. We extend the POSSUM MR simulator [44, 45], and combine it with an existing diffusion framework [46], in order to produce realistic DW-MR datasets with many of the artefacts typically seen in real data. Our analysis directly measures the important outcomes for correction strategies: the ability to correctly estimate the underlying displacement field for correction, and the ability to recover information lost from regions of signal compression. We also use the simulation framework to evaluate one of the most commonly used surrogate metrics for assessing susceptibility correction, the comparison of corrected datasets acquired with AP and LR phase-encoding, and use this surrogate to extend our comparison to real data. Finally, we quantify the increased variability in diffusion metrics caused by the dynamic susceptibility artefact. We hope that this work will enable researchers to make more carefully informed choices when designing their acquisition and processing pipelines. We further hope that it will raise awareness of the dynamic susceptibility artefact and encourage development of techniques for dealing with it, and to this end we make the code and datasets used in this work available at https://fsl.fmrib.ox.ac.uk/fsl/fslwiki/POSSUM.

## Methods

We first describe how we simulate DW-MR datasets with realistic susceptibility artefacts, suitable for assessing methods for their correction. We then describe both the experiment design and data simulated for these assessments.

### Simulation of DW-MR datasets

A simulation-based evaluation of correction methods requires realistic simulated DW-MR datasets with artefacts, along with a suitable ground truth. We chose to use POSSUM [44, 45] for our simulations because it is a highly realistic simulator that solves Bloch’s and Maxwell’s equations to generate complex data in *k*-space, ensuring that the images and their artefacts capture the key features of their real-world counterparts. A significant advantage of POSSUM in this context is its ability to model the interaction between the susceptibility artefact and motion which, to our knowledge, no other simulator is currently capable of. POSSUM is primarily an fMRI simulator, and so two modifications were made to enable the production of DW-MR datasets: the implementation of the spin-echo (SE) pulse sequence, and the incorporation into a recently proposed framework that enables the simulation of diffusion-weighted data [47]. Both of these modifications are described in S1 Appendix. Taken together, they enable a comprehensive framework, able to produce realistic DW-MR datasets with many of the artefacts typically seen in real data, including: susceptibility, eddy-currents, motion both between and during acquisitions of volumes, signal dropout, Gibbs ringing, chemical shift, ghosting, spiking and *T*_1_ history effects.

**Fig 1:**
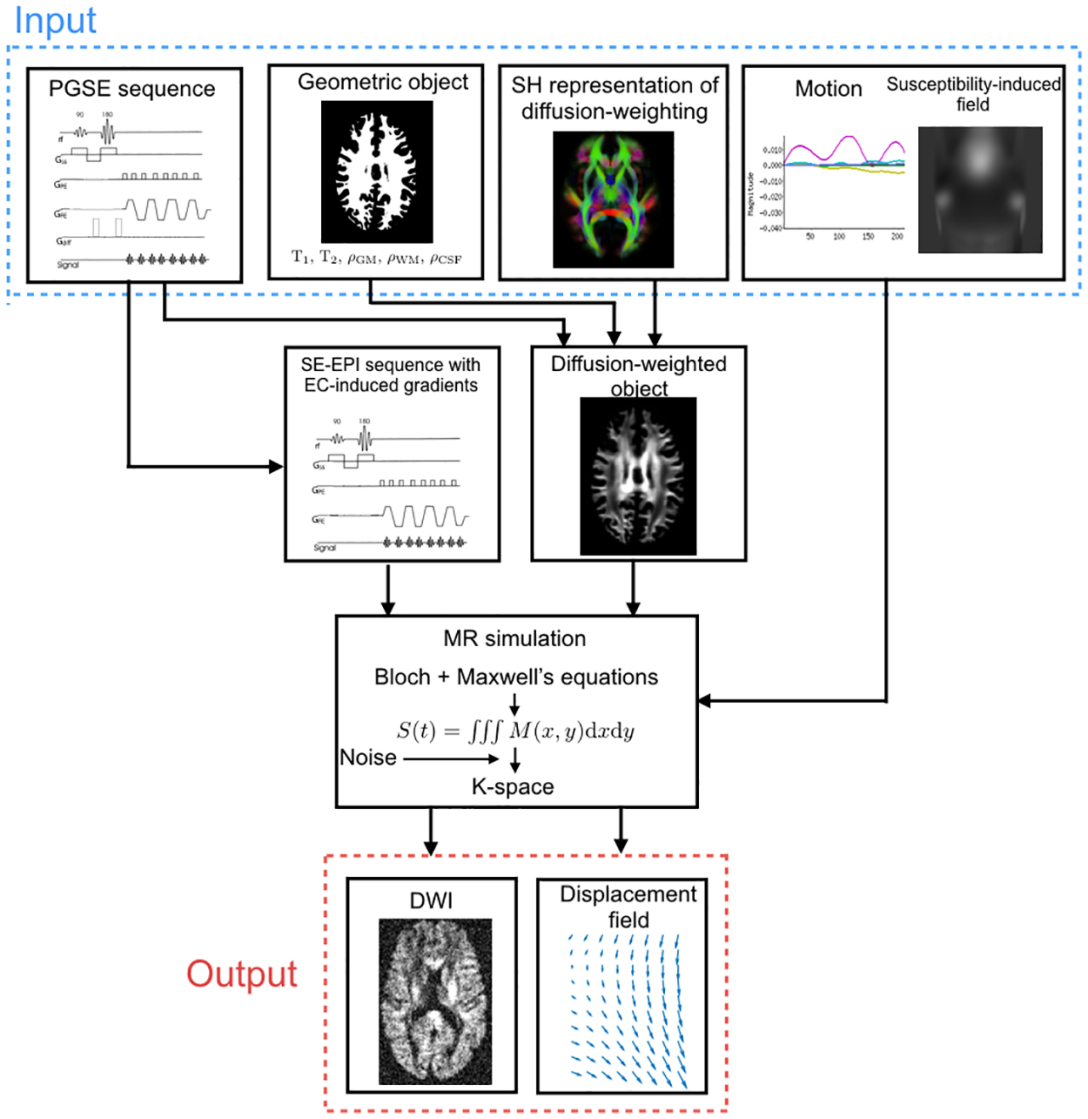
The pipeline for simulating DWIs.

Fig 1 shows the full simulation framework. In addition to DW-MR data, the framework outputs a displacement field, describing the geometric mapping of data distorted by artefacts (e.g. motion, eddy-currents and susceptibility) to a ground-truth space. This can be used to directly and quantitatively evaluate correction methods, by comparing their estimated displacement fields with the simulator ground truth.

Fig 1 shows that the framework requires a number of inputs. In this work the input object was created from a full-brain segmentation of the T1- and T2-weighted images from a single subject from the WU-Minn HCP dataset [48], using FSL’s FAST [49]. The representation of diffusion weighting was achieved using a voxel-wise spherical harmonic (SH) fit to the *b* = 1000 s/mm^2^ shell of the subject’s diffusion data [50] using order n = 8. In order to produce data with distortions similar to those seen in real data, the susceptibility-induced off-resonance field was obtained from a GRE field-mapping scan of the same subject, using the following steps (as recommended in the commonly used fsl_prepare_fieldmap script): 1) the magnitude GRE scan was masked; 2) the mask was eroded using a 5 mm box kernel 3) the fieldmap was estimated for all voxels within the mask using FSL’s fsl_prepare_fieldmap script; 4) the resultant fieldmap was smoothed using a 3D Gaussian with sigma equal to 5mm to remove noise and extrapolated to vary smoothly outside the domain of the eroded mask.

### Assessment of susceptibility correction

The aim of this work is to assess the performance and limitations of existing methods for correcting the susceptibility artefact. Not only does the susceptibility-induced field produce geometric distortions in the data, but when the head rotates around an axis non-parallel to that of the main *B*_0_ field, the susceptibility-induced field is altered and the artefact cannot be fully corrected using a field estimated before the head moved [10]. Whilst work has characterised [6] and attempted to correct [41, 42] this effect in fMRI, we are not aware of any available post-processing methods that address the issue in DW-MR. As a result we divide out analysis into three parts: in the first two, we compare existing methods for susceptibility correction on data with the susceptibility artefact but no head movement, using both simulated and real data. In the third, we characterise the impact of neglecting the movement-susceptibility interaction on the analysis on DW-MR data, using simulated data. In the following we describe the experiment design for each component of the assessment.

#### Assessment of existing techniques using simulated data

In this section we describe the comparison of existing methods for susceptibility correction on data with the susceptibility artefact but no head movement. In DW-MR the susceptibility artefact leads to geometric distortions of the data along the PE direction. The non-linear nature of these distortions mean they can cause redistribution of the signal which appears as either a compression or stretch. In regions of compression some information is lost, and additional information is required in order to recover the true signal. An ideal susceptibility correction method will both correctly estimate the underlying geometric distortions and the true original signal.

There are three main classes of post-processing technique used for correcting the susceptibility artefact. Registration based (RB) techniques non-linearly register the distorted data to a non-distorted structural target, often a T2-weighted image due to its similar contrast to the b = 0 volume. Fieldmap based (FMB) estimate the off-resonance field from a series of images with different echo times, and then use this field to predict the underlying displacement field needed to correct for geometric distortions. Both RB and FMB techniques provide only a first-order correction of the signal changes, achieved by modulating the corrected image by the Jacobian of the local displacement field. Multiple phase-encoding based techniques (MPB) use multiple images acquired with different phase-encoding directions, and thus with different distortions, in order to estimate the underlying field needed to correct the data. If only a single DWI is acquired with multiple PE directions, the technique enables just the estimation of the field used to correct the dataset and employs the same first-order correction of signal intensity that is possible using RB and FMB techniques. If the full dataset is additionally acquired with multiple PE, these methods offer the added potential to recover the information lost from regions of signal compression, because these regions will instead be expanded in the reversed PE dataset. We refer to this special case of the MPB technique as full multiple phase-encoding based (MPB/F).

**Fig 2:**
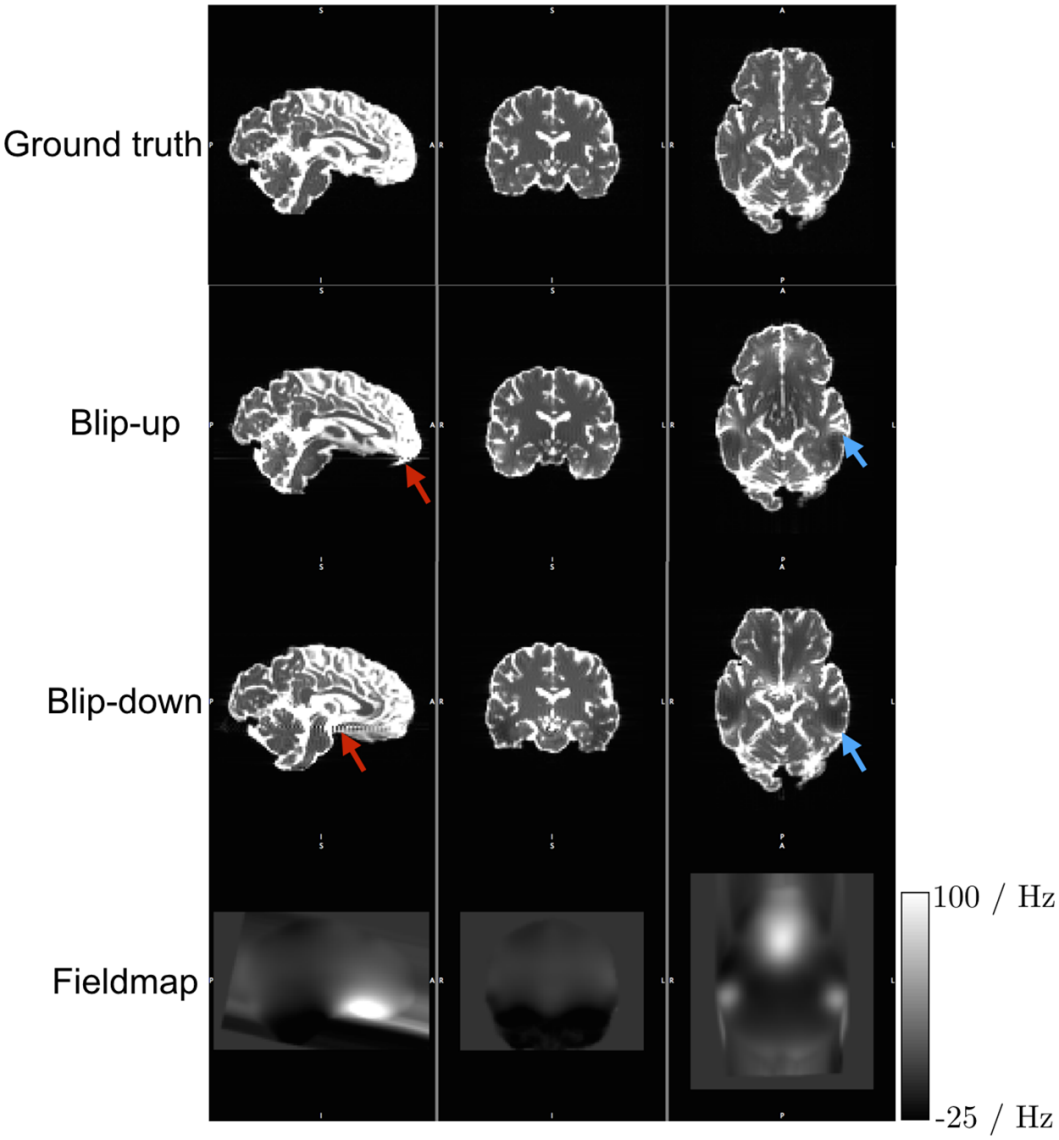
Simulated DWIs. Pairs of coloured arrows point to corresponding pairs of compression and expansion in the blip-up and blip-down images. The ‘streaking’ visible in the fieldmap is caused by the linear extrapolation to ensure a continuous field at the edge of the brain. The bounding box visible around the fieldmap is caused by its resampling into the space of POSSUM’s input object; this is not a problem for the simulation as the fieldmap is smooth and defined over all brain voxels in input object. Some Gibbs ringing is visible in the sagittal views of the distorted data—this is induced by sharp boundaries in regions of signal pile-up.

In order to assess these techniques, we simulated DW-MR datasets with susceptibility distortions, along with a T2-weighted structural image, field-mapping scans, and an additional DW-MR dataset with a reversed PE direction to enable application of the RB, FMB and MPB techniques, respectively. We designed the DW-MR datasets to contain levels of distortion similar to that found in recent high-end studies, such as the HCP [48] and UK Biobank, which forego the use of in-plane acceleration techniques (IPAT) in favour of SMS techniques, in order to characterise the ability of these techniques to correct data in the ‘worst case’ scenario. The DW-MR data, shown in Fig 2, was simulated with 32 volumes *b* = 1000 s/mm^2^ and 4 b = 0 volumes. We used a matrix size of 90×106, which was chosen along with the image voxel size (2 mm isotropic) and number of slices (68) to strike a balance between minimising computation time and ensuring full-brain coverage. The echo spacing was 1 ms, and no IPAT was used, leading to a PE bandwidth per pixel of 9.5 Hz, similar to values for data from the HCP project (9) and the HCP lifespan data acquired on a 3T Prisma scaner (10.4). Partial fourier was not used as POSSUM is currently unable to simulate it—this does not affect the level of susceptibility distortion in the data [51], but meant our TE of 109 ms is slightly higher than typical. K-space was apodized using a Hamming window, and no zero-filling was performed. Data was acquired with both posterior-anterior (PA) and anterior-posterior (AP) PE directions. Gaussian noise was added to the real and complex channels of the k-space data at two different levels, to produce datasets containing Rician noise with an average whole-brain SNRs of 40 and 20, as determined on the b = 0 volume—these represent the upper and lower bounds of SNR that we expect on modern scanners. Five realisations of each noise level were simulated, as well as a noise-free dataset. No other artefacts (e.g. eddy-currents, motion and concomitant fields) were included in the simulations. We also simulated a ground-truth set of DWIs, acquired with the same acquisition parameters but no input susceptibility field. The structural T2 was simulated with 1 mm isotropic resolution, dimensions 180×212×136 using a conventional spin-echo sequence with TE = 110 ms, TR = 2200 ms and a flip-angle of 90°. The field-mapping acquisition emulated the standard field-mapping scan found on a Siemens scanner, and involved the simulation of two gradient-echo images with the same voxel dimension and matrix size as the DW-MR scans, using a TR = 700 ms, flip-angle of 60° and TE values of 4.92 ms and 7.38 ms. Noise was added to these scans using the same standard deviation used in the DW-MR data, to simulate the same level of thermal noise in all datasets.

In this paper we tested a representative correction technique from each of the three classes. For the RB method we used the reg_f3d command from NiftyReg (Git commit bf926) [52], which uses a cubic b-spline deformation model. The first b = 0 volume was registered to the T2-weighted structural, and the estimated transform applied to each DWI. We used default settings for the registration but constrained the deformation field along the PE axis, emulating standard practice for susceptibility correction [16, 18, 53], and after experimentation set the bending energy term to 0.01. For the FMB methods we used the following steps: 1) mask the first magnitude GE image; 2) erode the mask by one voxel; 3) estimate the fieldmap for all voxels inside the mask using FSL’s PRELUDE (version 5.0.9) [54]; 4) apply the fieldmap to each DWI using FUGUE [55], smoothing the fieldmap using a 3D Gaussian kernel with sigma equal to 1 voxel (2 mm) as recommended in [6] and applying Jacobian modulation. For the MPB and MPB/F methods we used FSL’s TOPUP (version 5.0.9) [31], using the default supplied configuration file. For the MPB/F case we changed the resampling from least-squares resampling (LSR) to Jacobian, after noticing that LSR introduced some slight ‘ringing’ artefacts into our corrected data. TOPUP combines each PE pair by averaging them after Jacobian modulation. After correction each dataset was transformed into the same space by rigidly registering its b = 0 image to the noise-free, ground truth b = 0 image using a 6 degrees-of-freedom (DOF) transform with NiftyReg’s reg_aladin tool and then applying the estimated transform to each volume in the dataset.

The evaluation strategy is divided into three parts. Firstly, we assess the ability of each method to recover the correct underlying displacement field, and thus produce anatomically faithful data, by comparing each method’s estimated field to the ground truth field obtained using Eq 4 in S1 Appendix. Secondly, we assess the ability of each method to recover the correct intensity at each voxel by computing difference maps between the corrected and ground truth images. Finally we investigate the impact of correction quality on subsequent analysis by comparing diffusion tensor (DT) fits in both corrected datasets and ‘ground truth’ datasets, simulated free of artefacts.

In addition to the experiments described, we investigated a surrogate metric for correction quality that is often used to assess performance on real data: the comparison of corrected datasets that have been acquired with both AP and LR phase-encoding [18, 34]. The expectation is that the greater the correction quality, the greater the similarity between the corrected datasets. We aimed to evaluate whether this is a suitable surrogate for the most direct measure of correction quality, i.e. the displacement field error. To enable this experiment we simulated additional DW-MR data with LR and RL phase-encoding. These datasets were correcting using the same methods described above, and then compared to the corrected datasets acquired with AP and PA phase-encoding.

#### Assessment of existing techniques using real data

In this section we extend our evaluation of existing techniques to real data. To enable this evaluation we used the surrogate metric described in the previous section, the comparison of corrected datasets with both AP and LR phase-encoding. We used ten subjects from the developing HCP project [56]. We selected this dataset because it provides DW-MR data acquired with four PE directions: AP, PA, LR and RL, fieldmaps and structural data, enabling correction using all the methods used in this paper and evaluation using the surrogate metric. The data was acquired on a 3T Philips Achieva, consisting of a spherically optimized set of directions on 4 shells (b0: 20, b400: 64, b1000: 88, b2600: 128) split into four PE subsets. It was acquired using an acceleration of MB 4, SENSE factor 1.2 and partial fourier 0.86, TR/TE 3800/ 90 ms. The acquired resolution is 1.5x1.5 mm, 3 mm slices with 1.5 mm overlap, reconstructed to give data of resolution 1.17x1.17x1.5 mm. The T2-weighted image had TR/TE 12000/156 ms with a reconstructed resolution of 0.8 mm^3^. AP and LR datasets were corrected separately using the correction methods as described previously. Corrected b = 0 images were rigidly registered to a T2-weighted image using a 6 degrees-of-freedom (DOF) transform with NiftyReg’s reg_aladin tool and the similarity between AP and LR corrected images assessed.

#### Assessment of the susceptibility-movement interaction

In this section we describe the experiments performed to characterise the impact of the interaction between the susceptibility field and head motion. This is an effect that none of the commonly used post-processing correction strategies currently account for in DW-MR. To investigate the impact of this on analysis of data, we compare state-of-the-art correction on datasets simulated with and without a dynamic susceptibility field.

DW-MR data was simulated with the same parameters as in the previous section, using a full AP and PA acquisition totalling 72 volumes. A key difference to the previous section’s simulations is that here we calculated the field from an air-tissue segmentation of POSSUM’s input object using a perturbation method as described in [57]. This method is physically motivated, providing realistic fields, and provides a set of basis-functions that enable POSSUM to calculate how the susceptibility-induced field changes as the head moves. Movement was simulated during the data acquisition, using motion parameters measured from a healthy patient during an MRI exam, to emulate a scan with a normal level of motion. Movement was simulated to occur between the acquisition of volumes. A second dataset was simulated with the same set of parameters but the level of motion scaled up by a factor of three, to emulate a situation where a patient moves a lot. For all the simulations, the translation parameters were set to 0 as, on the assumption that the main field is entirely uniform, translations do not contribute to the dynamic susceptibility effect and their inclusion would require the imaging FOV to be increased, which would increase computation time. We also create an additional set of simulations to control for the effects of image interpolation on our correction. These control datasets were simulated assuming the static case where the susceptibility field remains unchanged as the head moves—this situation matches the assumptions of existing susceptibility correction techniques. All datasets were simulated without noise.

Each dataset was corrected to mimic ‘state-of-the-art’ correction, in which motion and the static portion of the susceptibility field are corrected for. Ground truth displacement fields for each volume were created from the motion parameters and the static portion of the field, both of which are known inputs to the simulation, and applied. Two sets of correction were performed: in the first, only the AP dataset was corrected, and in the second, joint correction of the AP and PA dataset was carried out to enable correction in regions of compression. The impact of residual distortions caused by the dynamic portion of the susceptibility field was demonstrated using errors in displacement fields, and the impact of the dynamic field on subsequent analysis was measured by characterising the errors in estimated FA values in corrected data.

## Results

### Assessment of techniques with simulated data

**Fig 3:**
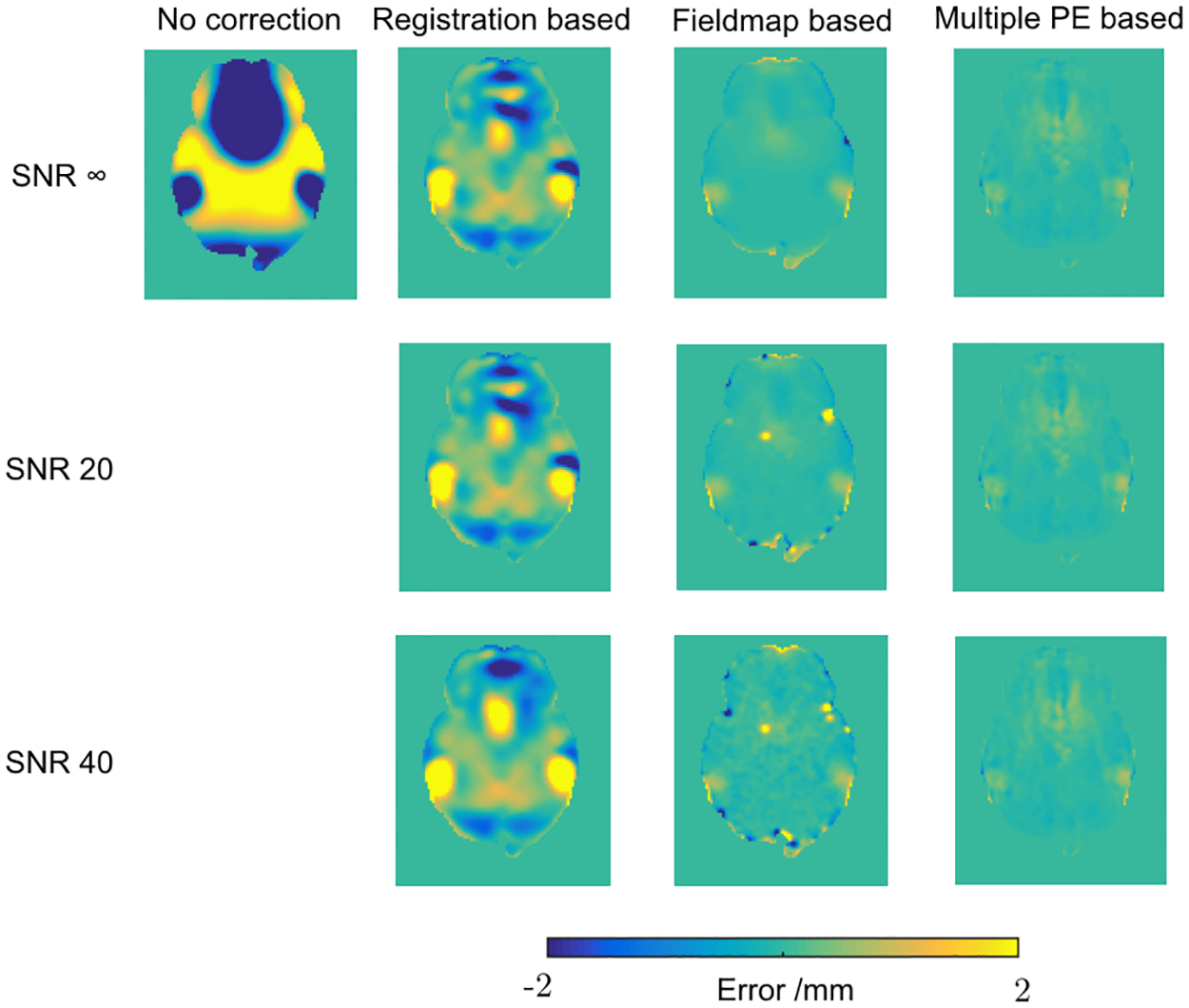
Displacement field errors. Error in displacement fields estimated by the three methods, assessed by subtraction from the ground truth field. One axial slice shown.

Fig 3 shows the errors in the displacement fields estimated by the three methods across a representative slice of the brain (the MPB and MPB/F methods use the same displacement field, estimated from just the b = 0 images with reversed PE). Full results for the five noise realisations are shown in Table 1. It is immediately clear that the RB method is unable to accurately estimate the underlying displacement field, whilst the FMB and MPB methods show better performance. The FMB method shows some errors in brain voxels that contain partial volume with air, around the edges of the brain, and these errors are introduced into brain voxels when the estimated fieldmap is smoothed. When the edge voxels are excluded using an eroded brain mask, the mean absolute errors per voxel reduce more for the FMB method than other methods (Table 2). The FMB’s difficulty estimating the field in edge voxels is exacerbated as the noise level increases, whilst the MPB method is relatively unaffected by noise.

**Table 1:** Mean of absolute errors in displacement field across the brain. Values shown are the mean across the five noise realisations, and errors are the standard deviation of the mean value for each noise realisation. Note that the multiple phase-encode results cover both MPB and MPB/F methods.

	Registration	Fieldmap	Multiple PE
SNR ∞	0.24	0.051	0.032
SNR 40	0.263 ± 0.011	0.071 ± 0.003	0.032 ± 0.000
SNR 20	0.272 ± 0.013	0.087 ± 0.002	0.036 ± 0.000

**Table 2:** The same metrics in Table 1 but calculated over an eroded brain mask. Values shown are the mean across the five noise realisations, and errors are the standard deviation of the mean value for each noise realisation.

	Registration	Fieldmap	Multiple PE
SNR ∞	0.24	0.024	0.029
SNR 40	0.249 ± 0.010	0.035 ± 0.001	0.029 ± 0.000
SNR 20	0.251 ± 0.012	0.048 ± 0.002	0.033 ± 0.000

**Fig 4:**
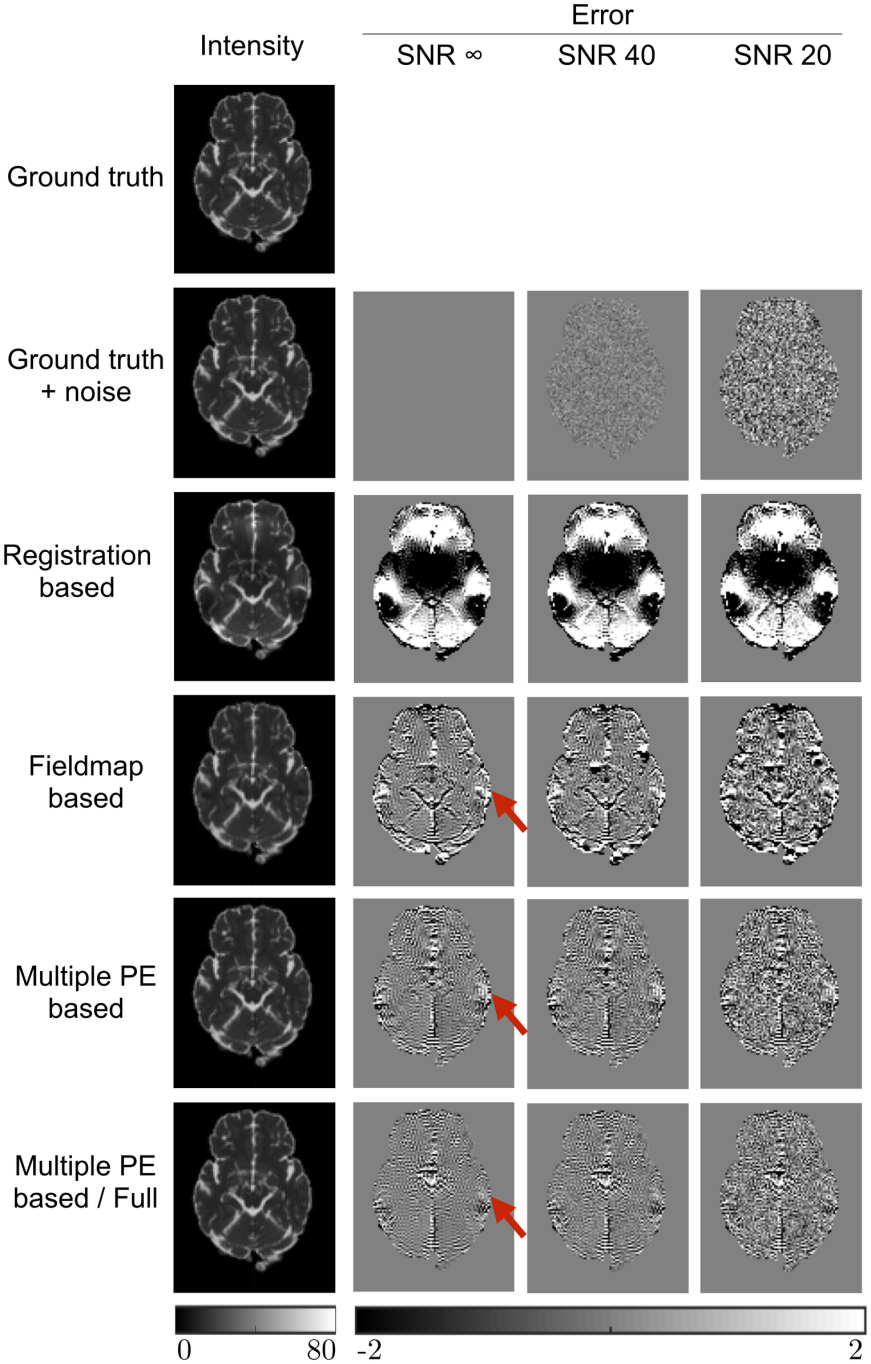
Errors in image intensity. b = 0 images after correction by each method, along with the ground truth images shown both with and without noise. Intensity images shown for the infinite SNR case. Error maps are obtained by subtraction from the noise-free ground truth image. Units are arbitrary signal units. Red arrows highlight a region of signal compression that can only be corrected by the MPB/F method. Note the MPB/F method uses twice as much data as the other methods, increasing its effective SNR.

The impact that the displacement field estimation has on the corrected data is demonstrated in Fig 4, which shows the b = 0 images after correction, along with error maps obtained by subtraction from the ground truth images. These results are shown in full in Table 3. The effects of the RB method’s poor estimation are clear in these results. The effect of the FMB method’s poor displacement field estimates in edge voxels is apparent here. Both the MPB and MPB/F methods show better results. The figure demonstrates a region of higher error in the FMB and MPB methods, due to their inability to recover the correct signal from a region of compression, where the MPB/F does better, due to its ability to resample from the corresponding expanded region in the reversed PE image. There is some slight ringing noticeable in the error maps, particularly for the MPB and MPB/F methods. This is Gibbs ringing present in the ground-truth b = 0 images, caused by the strong CSF rim. The corrected images have much reduced ringing because they have been smoothed by interpolation, so the ringing is visible upon subtraction. The ringing appears less visible for RB and FMB methods because it is obscured by larger errors caused by poorer correction.

**Table 3:** Absolute errors in image intensity, averaged across the brain for all b = 0 and DWI volumes. Values shown are the mean across the five noise realisations, and errors are the standard deviation of the mean value for each noise realisation. Units are arbitrary signal units.

		GT + noise	RB	FMB	MPB	MPB/F
SNR ∞	b = 0	0.00	2.41	1.36	0.73	0.59
	DWI	0.00	0.70	0.20	0.13	0.09
SNR 40	b = 0	0.23 ± 0.00	2.55 ± 0.04	1.71 ± 0.04	0.78 ± 0.00	0.62 ± 0.00
	DWI	0.23 ± 0.00	0.75 ± 0.01	0.35 ± 0.01	0.27 ± 0.00	0.19 ± 0.00
SNR 20	b = 0	0.70 ± 0.00	2.81 ± 0.03	2.09 ± 0.02	1.05 ± 0.00	0.80 ± 0.00
	DWI	0.70 ± 0.00	1.00 ± 0.00	0.69 ± 0.00	0.68 ± 0.00	0.49 ± 0.00

Table 3 demonstrates an additional advantage of the MPB/F method: the SNR boost obtained for each corrected image by resampling from two images, in effect averaging over the noise. It causes the mean errors for the DW volumes to be lower than the errors in the noisy ground-truth images. The results entangle two effects: the ability to recover information from regions of compression, and an SNR boost from having twice as much data. These can be disentangled by examining the SNR infinite case in Table 3, where the improvements from the MPB/F are solely due to improved signal recovery in regions of compression.

**Fig 5:**
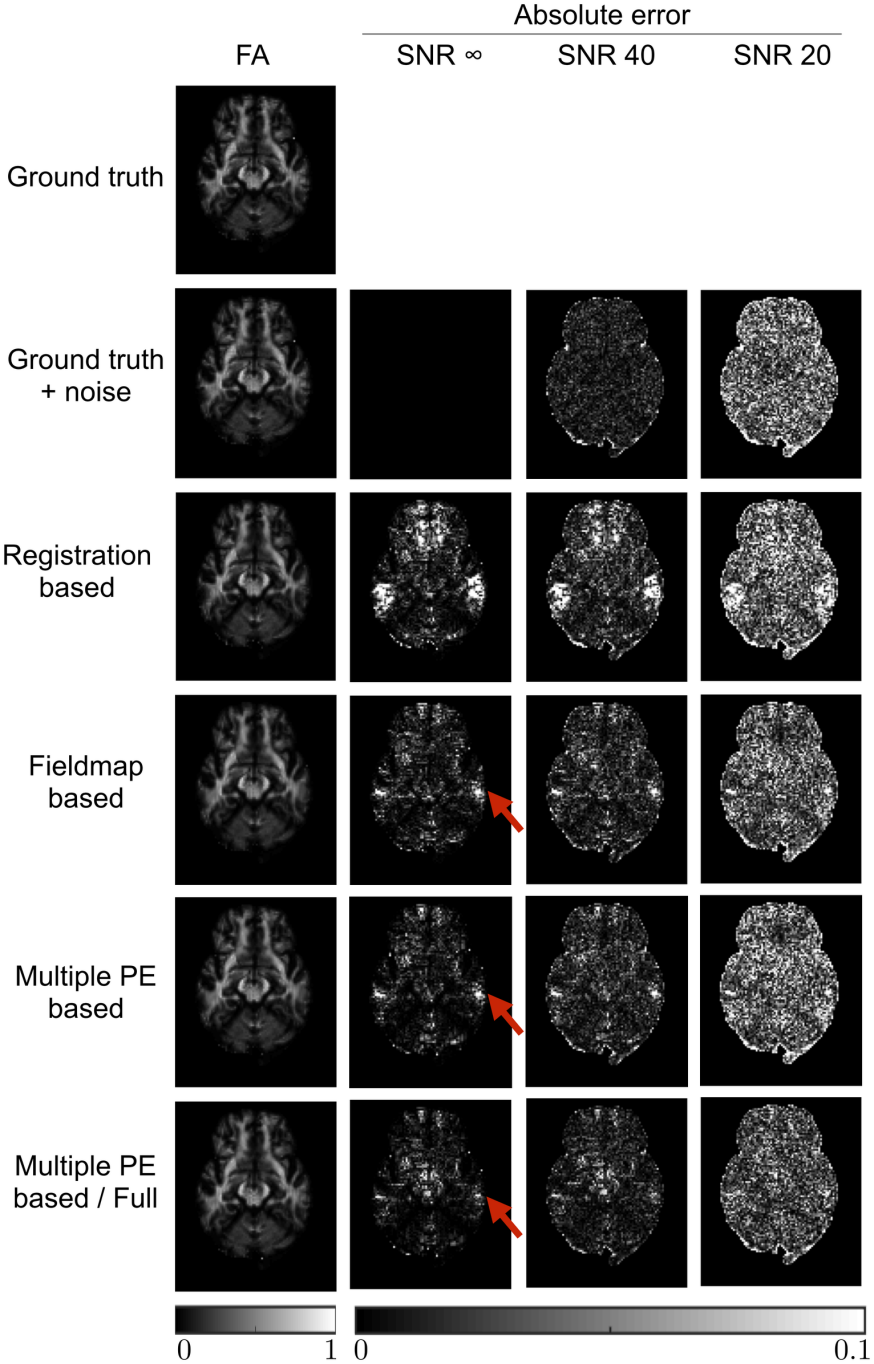
Errors in FA metrics. FA maps estimated from corrected and ground truth images, along with error maps obtained by subtraction from the noise-free ground truth estimate. FA map shown for SNR infinite case. Red arrows show regions of high error caused by signal pileup that could not be corrected by the RB and MPB methods, despite estimation of the correct displacement field. The MPB/F method is able to reduce errors in these regions. Note the MPB/F method uses twice as much data as the other methods, increasing its effective SNR.

Fig 5 show FA maps estimated from the corrected datasets for one slice of the brain, as well as their errors, with full results for FA, MD and the principle diffusion direction (V1) shown in Table 4. The downstream effect of information loss from areas of compression is appreciable in both the FMB and MPB maps, as is the ability of the MPB/F method to mitigate these errors. The SNR boost provided by the MPB/F method is visible in the figure, which shows some regions have lower error than the noisy ground truth maps, and also clear from the tabulated results. The results in Table 4 also demonstrate that the amount of smoothing introduced by a method can affect results. For noisy data the errors for corrected datasets are sometimes smaller than the error for the ‘ground truth + noise’ datasets, which reflects the noise-reducing effects of the local smoothing introduced upon correction. This effect is particularly strong for the FMB method, likely due to the smoothing of the estimated fieldmap during processing, causing it to produce smaller average errors for FA and V1 than the MPB method, despite MPB estimating the underlying displacement field more accurately. Dividing the errors according to the size of the underlying distortion (S1 Table) confirms that it is the smoothing that causes this; FMB tends to outperform MPB in areas of low distortion where errors are mostly controlled by the amount of noise, whilst MPB outperforms FMB in regions of large distortion where estimation of the correct underlying displacement field is important.

**Table 4:** Errors in diffusion metrics (FA, MD and the principle diffusion direction V1), averaged across the brain. Values shown are the mean across the five noise realisations. V1 errors were only calculated in voxels with a ground-truth FA >0.2. Errors (calculated as the standard deviation of the mean value for each noise realisation) not shown as they were all 0 to 3 decimal places.

		GT + noise	RB	FMB	MPB	MPB/F
SNR ∞	FA	0.000	0.017	0.014	0.010	0.008
	MD / 10^-3^ mm^2^ s^−1^	0.000	0.077	0.073	0.048	0.040
	V1 / degrees	0.000	3.602	1.693	1.008	0.687
SNR 40	FA	0.016	0.027	0.021	0.020	0.014
	MD / 10^-3^ mm^2^ s^−1^	0.022	0.087	0.090	0.055	0.046
	V1 / degrees	3.787	6.418	3.773	3.825	2.704
SNR 20	FA	0.051	0.053	0.044	0.049	0.033
	MD / 10^-3^ mm^2^ s^−1^	0.078	0.134	0.122	0.096	0.078
	V1 / degrees	11.580	11.775	9.872	10.931	7.744

**Fig 6:**
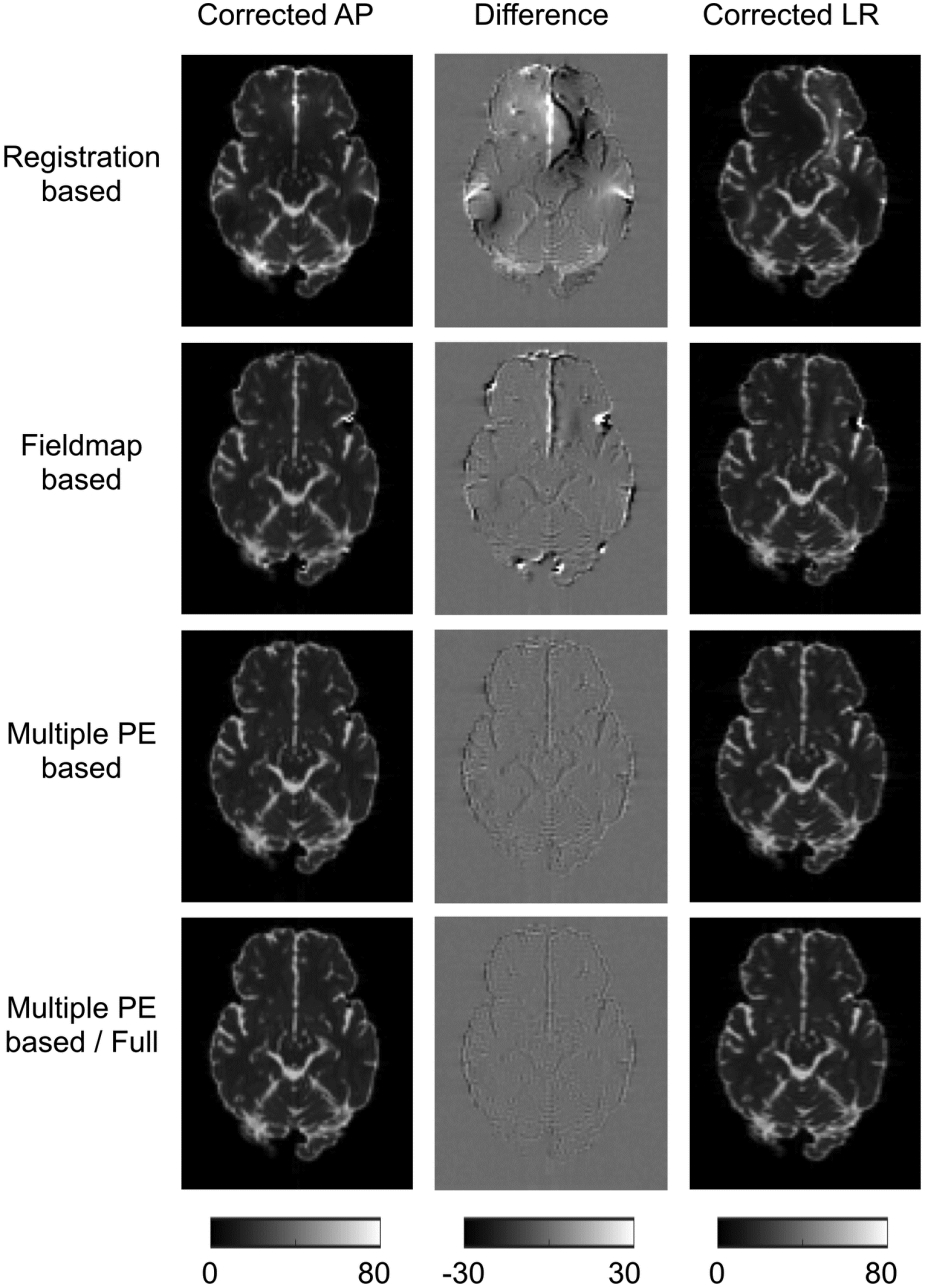
AP-LR comparison on simulated data. Figure shows corrected AP and LR b = 0 images, and the intensity difference between them. SNR = 40 dataset shown.

To investigate the suitability of AP-LR differences as a surrogate metric we plot the corrected AP and LR b = 0 images, and their differences, for a representative slice in Fig 6. The whole-brain mean of the intensity difference for b = 0 volumes was computed for every correction method, for each of the five noise realisations at each SNR—results are shown in Table 5. The results show the largest errors for the RB method with MPB and MPB/F performing the best, consistent with previous results. A two-sample t-test without assuming equal variance was performed between these values for each pair of correction methods; all differences were significant at the p<0.001 level. These results demonstrate that the surrogate metric shows the same ordering of correction ability as more direct metrics, such as error in displacement field, indicating that it can be a useful metric. However, it should be noted that the metric does not give the same contrast between methods as displacement field error. Errors in displacement fields show large differences between the RB and FMB methods, and a much smaller difference between FMB and MPB methods. The surrogate metric loses this contrast, indicating a roughly similar improvement going from RB and FMB methods as FMB to MPB methods. This is because there is not a simple relationship between displacement field error and intensity error; the size of intensity error depends on both the size and the location of the displacement error.

**Table 5:** Surrogate metrics for simulated data. Table shows whole-brain-mean intensity differences between AP and LR corrected datasets (units are arbitrary signal units). Errors are the standard deviation of the means over the five noise realisations. Metrics show statistically significant differences between all methods at the p<0.001 level.

	RB	FMB	MPB	MPB/F
SNR 40	3.791 ± 0.044	2.818 ± 0.047	1.904 ± 0.100	1.498 ± 0.025
SNR 20	3.976 ± 0.026	3.230 ± 0.041	1.989 ± 0.022	1.688 ± 0.069

### Assessment of techniques with real data

**Fig 7:**
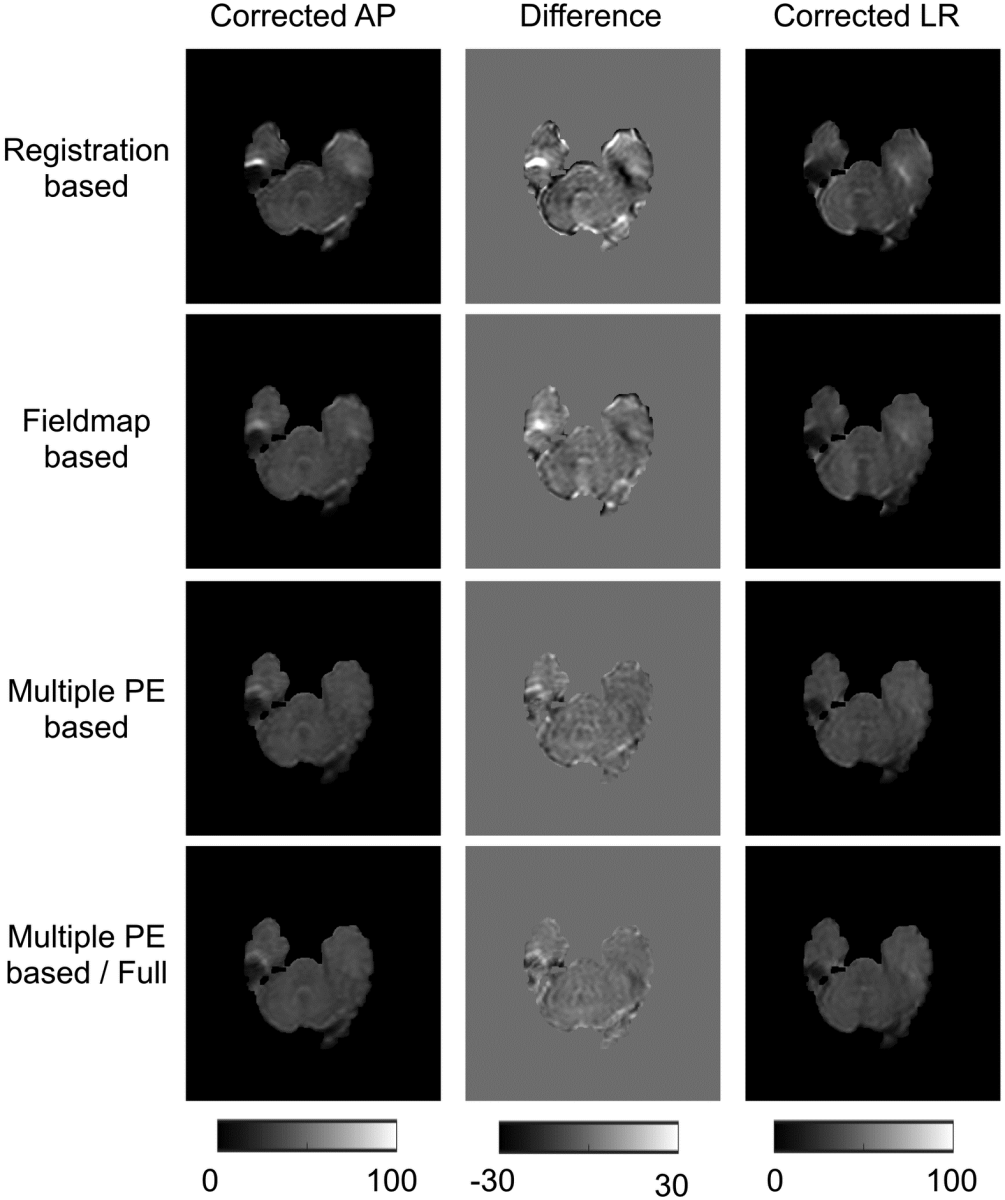
AP-LR comparison on real data. Figure shows corrected AP and LR b = 0 images, and the intensity difference between them.

Fig 7 shows the differences between AP and LR correction for real data. These results resemble findings in simulated data, indicating that RB methods perform the worst and MPB/F best. Table 6 reports the whole-brain mean of the intensity difference for b = 0 volumes. A paired t-test was performed between these intensity differences for each method, all differences were significant to p<0.001.

**Table 6:** Surrogate metrics for real data. Table shows whole-brain-mean intensity differences between AP and LR corrected datasets (units are arbitrary signal units). Errors are the standard deviation of the means over the ten subjects. Metrics show statistically significant differences between all methods at the p<0.001 level.

RB	FMB	MPB	MPB/F
6.463 ± 1.282	5.277 ± 1.278	3.579 ± 0.885	3.078 ± 0.965

### Interaction between susceptibility and movement

**Fig 8:**
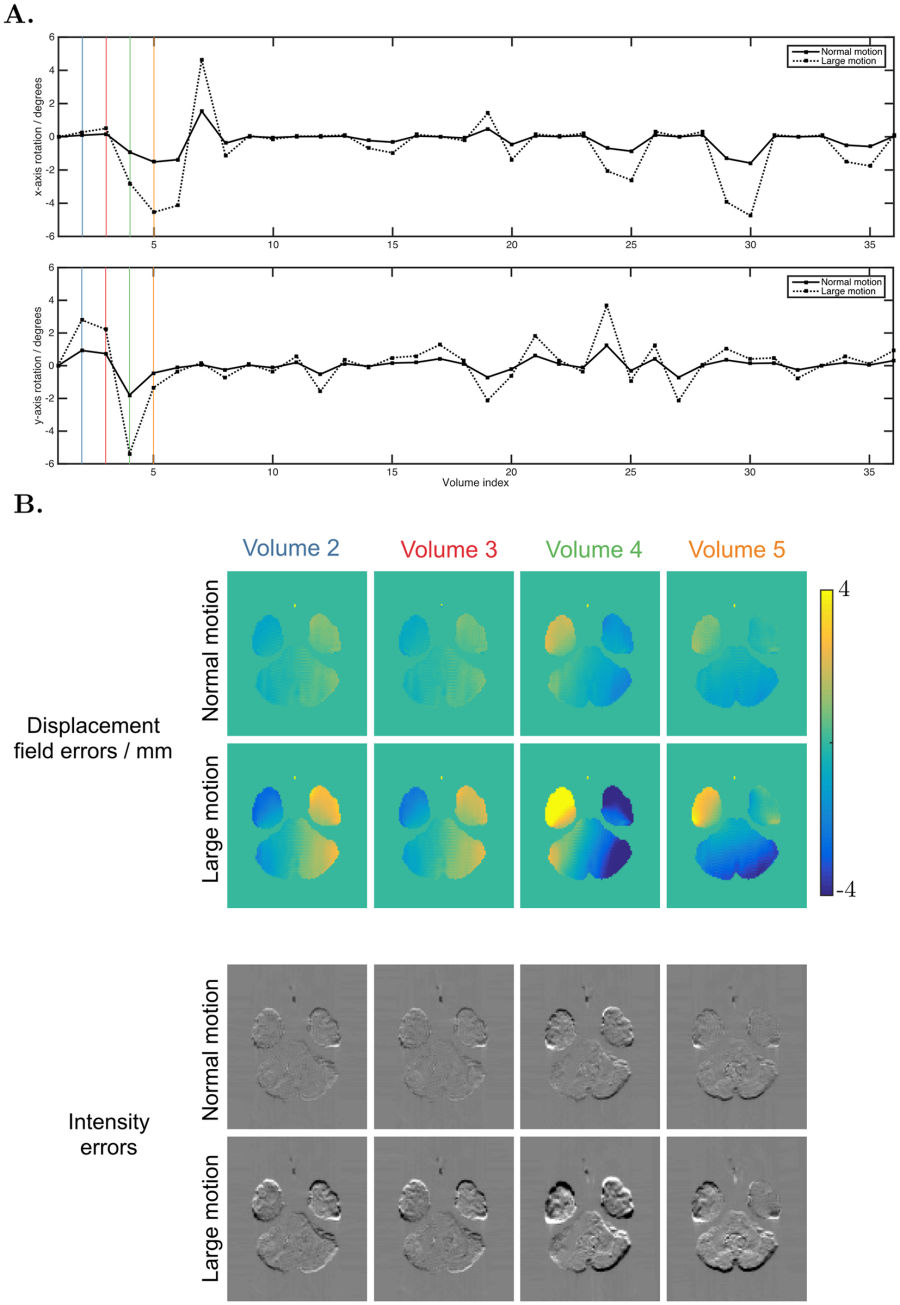
Susceptibility-movement interaction. A. The x- and y- rotation parameters used for the simulation of the first 36 volumes (z-rotations not shown because they do not contribute to the dynamic susceptibility effect, translations were all 0). The coloured vertical lines highlight the motion of the volumes depicted in plot B. B. Top two rows show the errors in displacement field caused by the dynamic portion of the susceptibility artefact, for volumes 2-5 of the acquisition—the motion these volumes experienced is highlighted with colour in plot A. Bottom two rows show the error in intensity of these volumes after they are corrected for motion and the static portion of the susceptibility field, obtained by subtraction from ground truth images.

Fig 8a shows the rotation parameters used for the simulations. Fig 8b shows the residual errors in the first four DWIs after correction for motion and static susceptibility. We found that a 5° rotation about the y-axis caused changes in the susceptibility field of up to 30 Hz, corresponding to distortions of up to 6 mm for the acquisition protocol used. This is slightly smaller than the field changes measured in real data—[10] found changes of 50 Hz for similar rotations at 3T—indicating our dynamic distortions are in a realistic range but may slightly underestimate the true size of the effect. Our simulations show a left-right asymmetry in dynamic displacement fields for rotations around *y* (Fig 8b, Volume 5) and left-right symmetry for rotations around *x* (Fig 8b, Volume 4) that matches observations made in real data [41].

**Fig 9:**
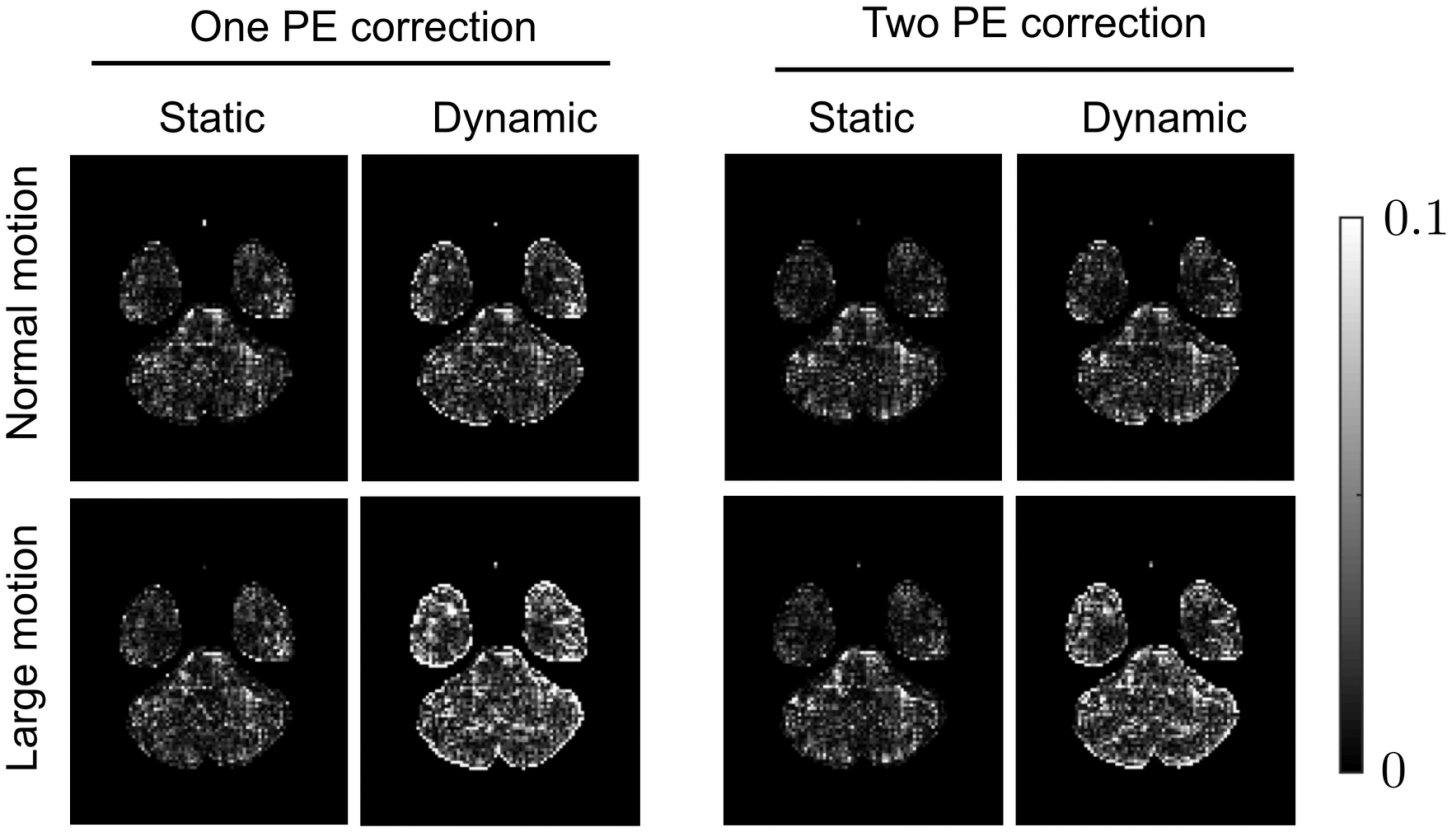
Errors introduced when failing to account for the susceptibility-movement interaction. Absolute errors in FA shown over one slice, for datasets corrected for motion and static susceptibility. Data in the static columns were simulated with only motion and static susceptibility artefacts, whilst the dynamic data contained motion and dynamic susceptibility. ‘One PE correction’ indicates only the AP data was used for correction, and ‘two PE correction’ indicates the AP and PA data were both use—note these are different from MPB and MPB/F, which are methods for both estimating and applying a displacement field, whilst in this case known ground-truth displacement fields have been applied.

**Fig 10:**
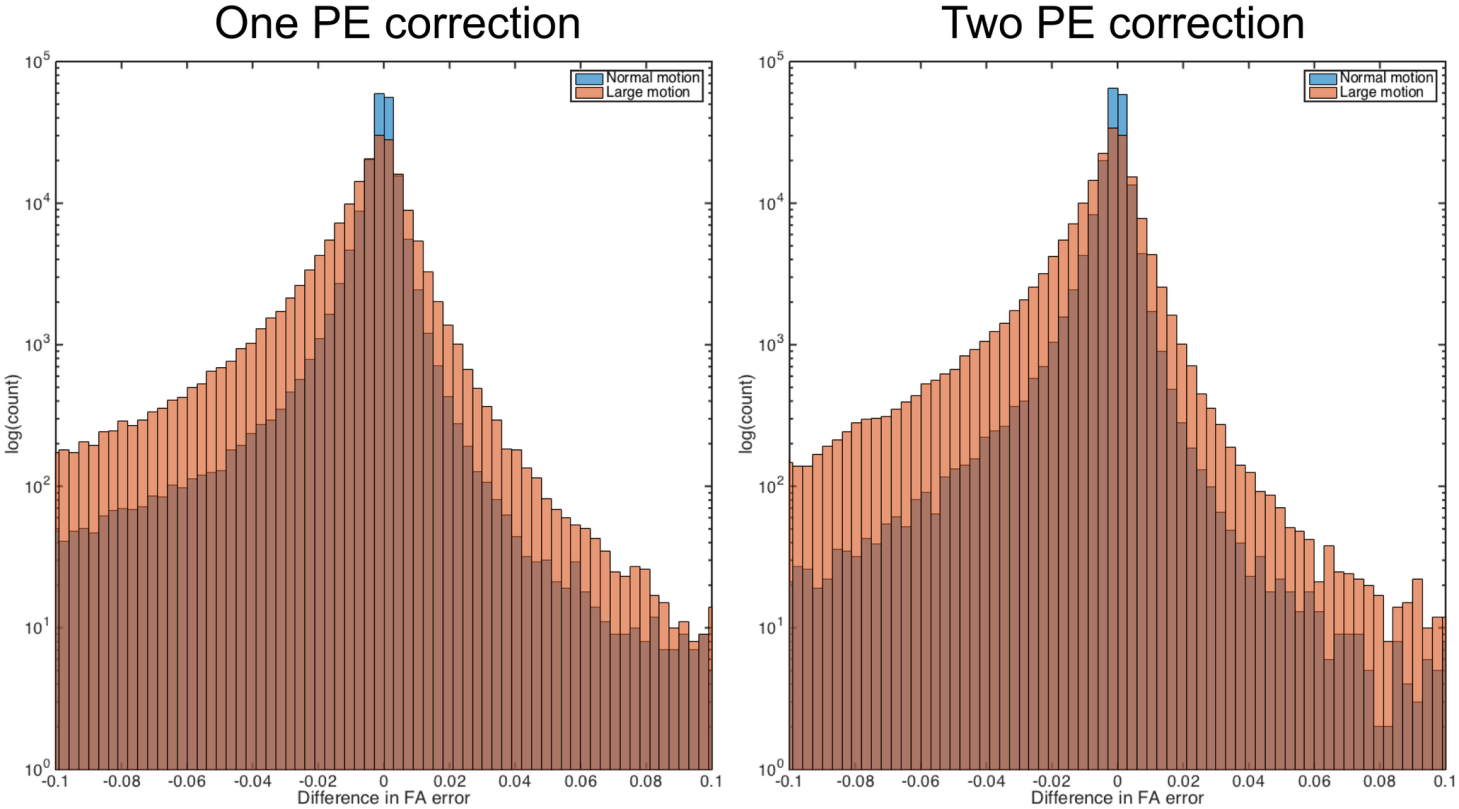
Error distribution. Histogram of the difference in absolute FA errors over the full brain, for datasets corrected for motion and static susceptibility: |ΔFA_static_| − |ΔFA_dynamic_|, so the heavy tail for negative values indicate higher errors for the dynamic case.

Fig 9 shows the errors in FA for corrected data across an example slice. Comparing data simulated with static and dynamic susceptibility fields, we see increased errors in the data with dynamic effects. The differences in static and dynamic errors across the full brain are shown in Fig 10. It is worth noting that the errors for the data with static susceptibility fields are non-zero, despite the data being noise free and corrected with the ground truth fields. These errors are introduced by interpolation effects, and introduce similar levels of error into both static and dynamic data, so it is the difference in error between these two sets of data that reveals the errors introduced by the dynamic susceptibility fields alone. The results show that the dynamic field increases FA errors especially for the case of the subject with larger motion. The effect of the dynamic field was slightly smaller for data corrected using both AP and PA volumes than data corrected using only the AP set.

## Discussion

In this paper we assessed the three main classes of technique used for correcting the susceptibility artefact, and investigated the impact of their inability to correct for the dynamic portion of the artefact. This work is particularly timely given that recent trends in acquisitions could lead to increased severity of the artefact, increasing the importance that the community has access to careful evaluations of the correction techniques available to them and their limitations. To enable our assessment, we extended an existing MR simulator and incorporated it into a framework that simulates realistic DW-MR datasets. This enabled us to directly and quantitatively assess the desired features of a susceptibility correction approach: the ability to both correct geometric distortions and recover the signal distribution lost in regions of compression. It further enabled us to carefully examine the impact that neglecting the dynamic susceptibility field has on analysis of diffusion data.

Our results showed that registration of distorted data to a structural T2-weighted volume was insufficient for fully correcting geometric distortions in the data. However, it offers improvement over performing no correction at all, and has the advantage that a structural volume is often acquired in a scan, making it suitable for retrospective studies where data required for FMB or MPB methods is not available (contrast inversion techniques may mean similar results can be obtained with a T1-weighted volume [23, 43]). FMB and MPB techniques performed better, though the FMB method demonstrated sensitivity to partial volume with air, particularly as the noise level increased. This occurred despite following standard practice of only estimating the field for voxels within an eroded mask, to exclude voxels with significant partial volume, and smoothly extrapolating the estimated field outside the domain of the mask. It has been reported that fitting a set of 3D discrete cosine transformations can lead to improvement [6], but to our knowledge this is not implemented in any available software packages. In addition to providing better correction than the FMB technique, the MPB method has the additional advantage [30] of being able to correct for concomitant fields which cause translations of slices far from the isocentre along the PE direction [58]. Concomitant fields cannot be measured by field-mapping scans and thus are not corrected by them. However, it should be noted that this sensitivity to concomitant fields is less of an issue for modern scanners with field strengths of 3T or above, as the effects of the fields scale with the inverse of the main magnetic field strength, and in a well-tuned system they are corrected for on the scanner itself [59].

Whilst FMB and MPB techniques were both able to estimate the underlying displacement field well, neither of them are able to provide the correct signal distribution in areas that have been compressed by the susceptibility artefact. This is an inherent limitation, which our results showed can be addressed using MPB/F. The downside of MPB/F is that each DWI needs to be acquired twice, doubling the acquisition time. Whether this is an acceptable trade-off is likely to be influenced by several factors such as the available acquisition time, desired analysis methods (e.g. compartment modelling, tractography), and the brain regions to be studied. It is worth noting that the MPB/F method increases SNR in the corrected images, so does not provide any time penalty if repeats are already being acquired to boost SNR through averaging.

Surrogate metrics are often used to evaluate correction quality on real data. Such metrics can sometimes be misleading; for example visual inspection of registration results, or use of similarity metrics, can be misleading because differences between source and reference images can appear small despite the underlying displacement field having been poorly estimated. In order to extend our analysis to real data, we used the simulation framework to validate one of the most promising surrogate metrics for correction quality, the difference between AP and LR corrected datasets. Our simulations demonstrate the metric can be a useful surrogate for the statistic of real interest—error in the ground truth displacement field. However, the results showed that the metric does not necessarily give the same contrast between methods as errors in displacement field, and it is possible to conceive of a situation where the metric gives a different ordering of correction quality than displacement field error (for example, a small displacement field error in a region with high intensity-contrast). We suggest that any surrogate metric must be interpreted cautiously, and ideally should be accompanied with supporting evidence from simulated data where a ground-truth is available.

Our findings are in agreement with the existing literature, which largely made use of real data. [18] used real data to compare RB and FMB methods. They found FMB methods outperformed RB methods in all regions affected by susceptibility artefacts. Interestingly, they found RB methods outperformed FMB in the superior few slices of the brain. This is likely because the data was acquired at 1.5T and thus suffered from measurable concomitant field-induced shifts in these slices, which FMB methods cannot correct for. [60] also used surrogate metrics on real data and found that FMB outperformed RB methods. [32] used real data to compare FMB and MPB/F techniques, finding MPB/F methods to be superior. [61] used simulations to compare RB, FMB and MPB/F (but not MPB) techniques. Whilst they used simpler simulations and less direct metrics to assess correction efficiency, they observed the same ordering of the technique’s effectiveness as we did. We are not aware of any published comparisons of FMB and MPB methods, likely the most relevant comparison for most researchers as they offer the potential for good correction of geometric distortions with only slightly increased acquisition times.

We also investigated the impact of failing to correct for the dynamic portion of the susceptibility field on the analysis of diffusion data. Our results highlight that even if a subject moves a little the dynamic field increases the errors in estimated diffusion metrics, but that the problem becomes much worse for subjects that move ‘a lot’. This result is important in the context of population studies, where a group that moves a lot (due to e.g. age, disease) is compared to a healthy control group that is likely to move less. Interestingly, we found correcting data using both AP and PA acquisitions did not further increase the errors introduced by dynamic susceptibility, and in fact marginally reduced them. We hypothesised an increase in error would occur because each corrected volume was created by combining information from two differentially distorted volumes, thus creating corrected data that was even more ‘wrong’ than data obtained simply resampling all the PA volumes. A potential explanation for the result is that the motion trace we used is characterised by small rotations for most volumes and occasional spikes of larger motion for some. Thus, for each PA (or AP) image with a large dynamic susceptibility component caused by large motion, its corresponding AP (or PA) volume is likely to be much less distorted, and so producing a corrected volume from the two leads to an averaging effect that serves to reduce the total amount of distortion in the corrected data.

To our knowledge, none of the commonly used post-processing schemes correct for the dynamic susceptibility artefact in DW-MR. There have been attempts to deal with the problem using real-time auto-shimming [39, 40] but these require non-standard pulse sequences and are only able to correct for the linear terms of the dynamic field. Acquisition of a field-map for each volume is also possible, at the expense of increased scan-time [62]. Registration of every DW-MR volume to an undistorted structural target has been suggested in the past [19], but is inappropriate given the differences in contrast between diffusion-weighted and structural volumes, especially for modern acquisitions which tend to make use of higher *b*-values with even more different contrast. One potential method for mitigating the artefact, available to users of the MPB/F method, would be to interleave the protocol such that the acquisition of each blip-up volume is immediately followed by the acquisition of its blip-down counterpart. The dynamic field could then be estimated for every pair of DWIs, on the assumption that there was negligible motion between the pair of DWIs. This would have the added benefit of reducing the issue of combining volumes with opposite PE direction which have different effective diffusion sensitisation, which happens when movement occurs between them. Our simulation assumed inter-volume movement, but in reality movement will occur during the acquisition of volumes. Intra-volume movement correction schemes do exist [63, 64], and would ideally be integrated into any technique that corrected for the dynamic susceptibility artefact.

There are some limitations to the work. The simulations used an off-resonance field estimated from a field-map as the input, which could introduce some circularity that favours the FMB method. It seems that this wasn’t an issue in this study, as FMB techniques were outperformed by MPB methods. The use of a real dataset to produce the input to the simulator enables the production of more realistic datasets than seen in other simulators [46], but raises the potential that the input may itself have residual artefacts in it that could affect the results, or could even bias our experiments in favour of the methods originally used to preprocess the input dataset.

We tested one available software implementation of each method; the large number available meant it was not practical to evaluate more. For FMB and MPB+MPB/F methods, we used the implementations most commonly used by the research community. We note that there are MPB methods [35] and MPB/F methods [34] that report better results than the method used in this work, TOPUP, and a comparison of these promising techniques may be the subject of future work. There are a number of available RB implementations, with no single one of them being clearly more popular than the others, each with a large number of parameters and settings that can be optimised, but we do not believe the choice of specific implementation will change our conclusions. This is because other published work comparing different RB methods to the one we tested [18, 61], support the conclusion that they are consistently outperformed by FMB and MPB+MPB/F methods. We encourage researchers to use our framework to benchmark the performance of their post-processing pipelines, and to this end we make the code and datasets used in this work available at https://fsl.fmrib.ox.ac.uk/fsl/fslwiki/POSSUM.

## Supporting information

S1 Appendix Modifications to POSSUM to enable DW-MR simulation. (PDF)

S1 Table Errors in diffusion metrics. As in Table 4, errors for FA, MD and the principle diffusion direction V1, but here divided into regions of interest based on the amount of distortion in the data. Values shown are the mean across the five noise realisations. V1 errors were only calculated in voxels with a ground-truth FA >0.2. Errors (calculated as the standard deviation of the mean value for each noise realisation) not shown as they were all 0 to 3 decimal places.
(PDF)

## References

[1] Assaf Y, Cohen Y. Inferring microstructural information of white matter from diffusion MRI. Diffusion MRI: From Quantitative Measurement to In-vivo Neuroanatomy. 2009;127 10.1016/B978-0-12-374709-9.00007-9

[2] Rose SE, Andrew L, Chalk JB. Gray and white matter changes in Alzheimer’s disease: a diffusion tensor imaging study. Journal of Magnetic Resonance Imaging. 2008;27(1):20–26. 10.1002/jmri.21231 18050329

[3] Weston PS, Simpson IJ, Ryan NS, Ourselin S, Fox NC. Diffusion imaging changes in grey matter in Alzheimer’s disease: a potential marker of early neurodegeneration. Alzheimer’s research & therapy. 2015;7(1):47 10.1186/s13195-015-0132-3 PMC4487800 26136857

[4] Uğurbil K, Xu J, Auerbach EJ, Moeller S, Vu AT, Duarte-Carvajalino JM, et al Pushing spatial and temporal resolution for functional and diffusion MRI in the Human Connectome Project. Neuroimage. 2013;80:80–104. 10.1016/j.neuroimage.2013.05.012 23702417 PMC3740184

[5] Turner R, Le Bihan D. Single-shot diffusion imaging at 2.0 Tesla. Journal of Magnetic Resonance (1969). 1990;86(3):445–452. 10.1016/0022-2364(90)90023-3

[6] Hutton C, Bork A, Josephs O, Deichmann R, Ashburner J, Turner R. Image distortion correction in fMRI: A quantitative evaluation. Neuroimage. 2002;16(1):217–240. 10.1006/nimg.2001.1054 11969330

[7] Andersson JL, Richter M, Richter W, Skare S, Nunes RG, Robson MD, et al. Effects of susceptibility distortions on tractography. In: Proceedings of International Society of Magnetic Resonance in Medicine. vol. 11; 2004. p. 87.

[8] Embleton KV, Haroon HA, Morris DM, Ralph MAL, Parker GJ. Distortion correction for diffusion-weighted MRI tractography and fMRI in the temporal lobes. Human brain mapping. 2010;31(10):1570–1587. 10.1002/hbm.20959 20143387 PMC6870737

[9] Irfanoglu MO, Walker L, Sarlls J, Marenco S, Pierpaoli C. Effects of image distortions originating from susceptibility variations and concomitant fields on diffusion MRI tractography results. NeuroImage. 2012;61(1):275–88. 10.1016/j.neuroimage.2012.02.054 22401760 PMC3653420

[10] Jezzard P, Clare S. Sources of Distortions in Functional MRI Data. Human Brain Mapping. 1999;8(May):80–85. 10.1002/(SICI)1097-0193(1999)8:2/3<80::AID-HBM2>3.0.CO;2-C 10524596 PMC6873315

[11] Moeller S, Yacoub E, Olman CA, Auerbach E, Strupp J, Harel N, et al Multiband multislice GE-EPI at 7 tesla, with 16-fold acceleration using partial parallel imaging with application to high spatial and temporal whole-brain fMRI. Magnetic Resonance in Medicine. 2010;63(5):1144–1153. 10.1002/mrm.22361 20432285 PMC2906244

[12] Setsompop K, Gagoski BA, Polimeni JR, Witzel T, Wedeen VJ, Wald LL. Blipped-controlled aliasing in parallel imaging for simultaneous multislice echo planar imaging with reduced g-factor penalty. Magnetic Resonance in Medicine. 2012;67(5):1210–1224. 10.1002/mrm.23097 21858868 PMC3323676

[13] Sotiropoulos SN, Jbabdi S, Xu J, Andersson JL, Moeller S, Auerbach EJ, et al Advances in diffusion MRI acquisition and processing in the Human Connectome Project. NeuroImage. 2013;80:125–143. 10.1016/j.neuroimage.2013.05.057 23702418 PMC3720790

[14] Miller KL, Alfaro-Almagro F, Bangerter NK, Thomas DL, Yacoub E, Xu J, et al Multimodal population brain imaging in the UK Biobank prospective epidemiological study. Nature Neuroscience. 2016;. 10.1038/nn.4393 PMC5086094 27643430

[15] Hughes EJ, Winchman T, Padormo F, Teixeira R, Wurie J, Sharma M, et al A dedicated neonatal brain imaging system. Magnetic Resonance in Medicine. 2016;. 10.1002/mrm.26462 27643791 PMC5516134

[16] Studholme C, Constable R Todd, Duncan JS. Accurate alignment of functional EPI data to anatomical MRI using a physics-based distortion model. IEEE Transactions on Medical Imaging. 2000;19(11):1115–1127. 10.1109/42.896788 11204849

[17] Kybic J, Thévenaz P, Nirkko A, Unser M. Unwarping of unidirectionally distorted EPI images. IEEE Transactions on Medical Imaging. 2000;19(2):80–93. 10.1109/42.836368 10784280

[18] Wu M, Chang LC, Walker L, Lemaitre H, Barnett aS, Marenco S, et al. Comparison of EPI distortion correction methods in diffusion tensor MRI using a novel framework. Medical image computing and computer-assisted intervention: MICCAI International Conference on Medical Image Computing and Computer-Assisted Intervention. 2008;11:321–329. 10.1007/978-3-540-85990-1_39 PMC4819327 18982621

[19] Ardekani S, Sinha U. Geometric distortion correction of high-resolution 3 T diffusion tensor brain images. Magnetic Resonance in Medicine. 2005;54(5):1163–1171. 10.1002/mrm.20651 16187289

[20] Tao R, Fletcher PT, Gerber S, Whitaker RT. A variational image-based approach to the correction of susceptibility artifacts in the alignment of diffusion weighted and structural MRI. In: International Conference on Information Processing in Medical Imaging. Springer; 2009. p. 664–675. 10.1007/978-3-642-02498-6_55 PMC4031680 19694302

[21] Gholipour A, Kehtarnavaz N, Scherrer B, Warfield SK. On the accuracy of unwarping techniques for the correction of susceptibility-induced geometric distortion in magnetic resonance Echo-planar images. In: Engineering in Medicine and Biology Society, EMBC, 2011 Annual International Conference of the IEEE. IEEE; 2011. p. 6997–7000. 10.1109/IEMBS.2011.6091769 PMC3675776 22255949

[22] Irfanoglu MO, Walker L, Sammet S, Pierpaoli C, Machiraju R. Susceptibility distortion correction for echo planar images with non-uniform B-spline grid sampling: A diffusion tensor image study. Lecture Notes in Computer Science (including subseries Lecture Notes in Artificial Intelligence and Lecture Notes in Bioinformatics). 2011;6892 LNCS(PART 2):174–181. 10.1007/978-3-642-23629-7_22 21995027

[23] Bhushan C, Choi S, Joshi Aa, Haldar JP, Shattuck DW, Leahy RM. Co-registration and distortion correction of diffusion and anatomical images based on inverse contrast normalization. NeuroImage. 2014; p. 30–33. 10.1016/j.neuroimage.2015.03.050 PMC4461504 25827811

[24] Jezzard P, Balaban RS. Correction for geometric distortion in echo planar images from B0 field variations. Magnetic resonance in medicine: official journal of the Society of Magnetic Resonance in Medicine / Society of Magnetic Resonance in Medicine. 1995;34(1):65–73. 10.1002/mrm.1910340111 7674900

[25] Reber PJ, Wong EC, Buxton RB, Frank LR. Correction of off resonance related distortion in echo planar imaging using EPI based field maps. Magn Reson Med. 1998;39:328–330. 10.1002/mrm.1910390223 9469719

[26] Yeo DTB, Chenevert TL, Fessler JA, Kim B. Zero and first-order phase shift correction for field map estimation with dual-echo GRE using bipolar gradients. Magnetic Resonance Imaging. 2007;25(9):1263–1271. 10.1016/j.mri.2007.02.001 17442524 PMC2795570

[27] Funai AK, Fessler JA, Yeo DTB, Noll DC, Olafsson VT. Regularized field map estimation in MRI. IEEE Transactions on Medical Imaging. 2008;27(10):1484–1494. 10.1109/TMI.2008.923956 18815100 PMC2856353

[28] Matakos A, Balter J, Cao Y. Estimation of geometrically undistorted B0 inhomogeneity maps. Physics in Medicine and Biology. 2014;59(17):4945–4959. 10.1088/0031-9155/59/17/4945 25109506 PMC4159702

[29] Chang H, Fitzpatrick JM. A technique for accurate magnetic resonance imaging in the presence of field inhomogeneities. IEEE Transactions on medical imaging. 1992;11(3):319–329. 10.1109/42.158935 18222873

[30] Morgan PS, Bowtell RW, McIntyre DJ, Worthington BS. Correction of spatial distortion in EPI due to inhomogeneous static magnetic fields using the reversed gradient method. Journal of magnetic resonance imaging. 2004;19(4):499–507. 10.1002/jmri.20032 15065175

[31] Andersson JLR, Skare S, Ashburner J. How to correct susceptibility distortions in spin-echo echo-planar images: Application to diffusion tensor imaging. NeuroImage. 2003;20:870–888. 10.1016/S1053-8119(03)00336-7 14568458

[32] Holland D, Kuperman JM, Dale AM. Efficient Correction of Inhomogeneous Static Magnetic Field- Induced Distortion in Echo Planar Imaging. NeuroImage. 2011;50(1):1–18. 10.1016/j.neuroimage.2009.11.044 PMC2819607 19944768

[33] Ruthotto L, Mohammadi S, Heck C, Modersitzki J, Weiskopf N. Hyperelastic susceptibility artifact correction of DTI in SPM. Informatik aktuell. 2013; p. 344–349.

[34] Irfanoglu MO, Modi P, Nayak A, Hutchinson EB, Sarlls J, Pierpaoli C. DR-BUDDI (Diffeomorphic Registration for Blip-Up blip-Down Diffusion Imaging) method for correcting echo planar imaging distortions. NeuroImage. 2015;106:284–299. 10.1016/j.neuroimage.2014.11.042 25433212 PMC4286283

[35] Hedouin R, Commowick O, Bannier E, Scherrer B, Taquet M, Warfield S, et al Block-Matching Distortion Correction of Echo-Planar Images With Opposite Phase Encoding Directions. IEEE Transactions on Medical Imaging. 2017;0062(c):1–1. 10.1109/TMI.2016.2646920 28092527

[36] Robson MD, Gore JC, Constable RT. Measurement of the point spread function in MRI using constant time imaging. Magnetic resonance in medicine: official journal of the Society of Magnetic Resonance in Medicine / Society of Magnetic Resonance in Medicine. 1997;38(12):733–740. 10.1002/mrm.1910380509 9358447

[37] Zeng H, Constable RT. Image distortion correction in EPI: Comparison of field mapping with point spread function mapping. Magnetic Resonance in Medicine. 2002;48(1):137–146. 10.1002/mrm.10200 12111941

[38] Zaitsev M, Hennig J, Speck O. Point spread function mapping with parallel imaging techniques and high acceleration factors: Fast, robust, and flexible method for echo-planar imaging distortion correction. Magnetic Resonance in Medicine. 2004;52(5):1156–1166. 10.1002/mrm.20261 15508146

[39] Ward HA, Riederer SJ, Jack CR. Real-time autoshimming for echo planar timecourse imaging. Magnetic Resonance in Medicine. 2002;48(5):771–780. 10.1002/mrm.10259 12417991

[40] Alhamud A, Taylor PA, van der Kouwe AJW, Meintjes EM. Real-time measurement and correction of both B0 changes and subject motion in diffusion tensor imaging using a double volumetric navigated (DvNav) sequence. NeuroImage. 2016;126:60–71. 10.1016/j.neuroimage.2015.11.022 26584865 PMC4733594

[41] Andersson JL, Hutton C, Ashburner J, Turner R, Friston K. Modeling geometric deformations in EPI time series. NeuroImage. 2001;13(5):903–919. 10.1006/nimg.2001.0746 11304086

[42] Hutton C, Andersson J, Deichmann R, Weiskopf N. Phase informed model for motion and susceptibility. Human Brain Mapping. 2013;34(11):3086–3100. 10.1002/hbm.22126 22736546 PMC6870252

[43] Taylor PA, Alhamud A, van der Kouwe A, Saleh MG, Laughton B, Meintjes E. Assessing the performance of different DTI motion correction strategies in the presence of EPI distortion correction. Human Brain Mapping. 2016;37(12):4405–4424. 10.1002/hbm.23318 27436169 PMC5118068

[44] Drobnjak I, Gavaghan D, Süli E, Pitt-Francis J, Jenkinson M. Development of a functional magnetic resonance imaging simulator for modeling realistic rigid-body motion artifacts. Magnetic Resonance in Medicine. 2006;56(2):364–380. 10.1002/mrm.20939 16841304

[45] Drobnjak I, Pell GS, Jenkinson M. Simulating the effects of time-varying magnetic fields with a realistic simulated scanner. Magnetic Resonance Imaging. 2010;28(7):1014–1021. 10.1016/j.mri.2010.03.029 20418038

[46] Graham MS, Drobnjak I, Zhang H. Realistic simulation of artefacts in diffusion MRI for validating post-processing correction techniques. NeuroImage. 2016;125:1079–1094. 10.1016/j.neuroimage.2015.11.006 26549300

[47] Graham MS, Drobnjak I, Zhang H. A Simulation Framework for Quantitative Validation of Artefact Correction in Diffusion MRI. In: Information Processing in Medical Imaging. vol. 9123; 2015. p. 638–649. Available from: http://link.springer.com/10.1007/978-3-319-19992-4{_}50. 10.1007/978-3-319-19992-4_50 26221709

[48] Van Essen DC, Ugurbil K, Auerbach E, Barch D, Behrens T, Bucholz R, et al The Human Connectome Project: a data acquisition perspective. Neuroimage. 2012;62(4):2222–2231. 10.1016/j.neuroimage.2012.02.018 22366334 PMC3606888

[49] Zhang Y, Brady M, Smith S. Segmentation of brain MR images through a hidden Markov random field model and the expectation-maximization algorithm. Medical Imaging, IEEE Transactions on. 2001;20(1):45–57. 10.1109/42.906424 11293691

[50] Alexander DC, Barker GJ, Arridge SR. Detection and modeling of non-Gaussian apparent diffusion coefficient profiles in human brain data. Magnetic Resonance in Medicine. 2002;48:331–340. 10.1002/mrm.10209 12210942

[51] Andersson JLR. Geometric Distortions in Diffusion MRI. Second edi ed. Elsevier; 2013 Available from: 10.1016/B978-0-12-396460-1.00004-4.

[52] Modat M, Ridgway GR, Taylor ZA, Lehmann M, Barnes J, Hawkes DJ, et al Fast free-form deformation using graphics processing units. Computer methods and programs in biomedicine. 2010;98(3):278–284. 10.1016/j.cmpb.2009.09.002 19818524

[53] Bhushan C, Joshi AA, Leahy RM, Haldar JP. Improved B0-distortion correction in diffusion MRI using interlaced q-space sampling and constrained reconstruction. Magnetic Resonance in Medicine. 2014;72(5):1218–1232. 10.1002/mrm.25026 24464424 PMC4017008

[54] Jenkinson M. Fast, automated, N-dimensional phase-unwrapping algorithm. Magnetic Resonance in Medicine. 2003;49(1):193–197. 10.1002/mrm.10354 12509838

[55] Smith SM, Jenkinson M, Woolrich MW, Beckmann CF, Behrens TEJ, Johansen-Berg H, et al Advances in functional and structural MR image analysis and implementation as FSL. In: NeuroImage. vol. 23; 2004 10.1016/j.neuroimage.2004.07.051 15501092

[56] Hughes E, Grande LC, Murgasova M, Hutter J, Price A, Gomes AS, et al. The Developing Human Connectome: announcing the first release of open access neonatal brain imaging. OHBM. 2017;.

[57] Jenkinson M, Wilson JL, Jezzard P. Perturbation method for magnetic field calculations of nonconductive objects. Magnetic Resonance in Medicine. 2004;52(3):471–477. 10.1002/mrm.20194 15334564

[58] Zhou XJ, Du YP, Bernstein MA, Reynolds HG, Maier JK, Polzin JA. Concomitant magnetic-field-induced artifacts in axial echo planar imaging. Magnetic Resonance in Medicine. 1998;39(4):596–605. 10.1002/mrm.1910390413 9543422

[59] Jones DK. Image Distortion and Its Correction in Diffusion MRI In: Diffusion MRI; 2009.

[60] Wang S, Peterson DJ, Gatenby JC, Li W, Grabowski TJ, Madhyastha TM. Evaluation of Field Map and Nonlinear Registration Methods for Correction of Susceptibility Artifacts in Diffusion MRI. Frontiers in Neuroinformatics. 2017;11:17 10.3389/fninf.2017.00017 28270762 PMC5318394

[61] Esteban O, Daducci A, Caruyer E, O’Brien K, Ledesma-Carbayo MJ, Bach-Cuadra M, et al. Simulation-based evaluation of susceptibility distortion correction methods in diffusion mri for connectivity analysis. 2014 IEEE 11th International Symposium on Biomedical Imaging, ISBI 2014. 2014; p. 738–741.

[62] Roopchansingh V, Cox RW, Jesmanowicz A, Ward BD, Hyde JS. Single-shot magnetic field mapping embedded in echo-planar time-course imaging. Magnetic Resonance in Medicine. 2003;50(4):839–843. 10.1002/mrm.10587 14523971

[63] Marami B, Salehi SSM, Afacan O, Scherrer B, Rollins CK, Yang E, et al Temporal slice registration and robust diffusion-tensor reconstruction for improved fetal brain structural connectivity analysis. NeuroImage. 2017;. 10.1016/j.neuroimage.2017.04.033 28433624 PMC5548611

[64] Andersson JLR, Graham MS, Drobnjak I, Zhang H, Filippini N, Bastiani M. Towards a comprehensive framework for movement and distortion correction of diffusion MR images: Within volume movement. NeuroImage. 2017;152(November 2016):450–466. 10.1016/j.neuroimage.2017.02.085 28284799 PMC5445723

