# Supplementary material for: Quantitative assessment of the susceptibility artefact and its interaction with motion in diffusion MRI
Source: PLoS One. 2017 Oct 2;12(10):e0185647. doi: 10.1371/journal.pone.0185647 (PMC5624609; doi:10.1371/journal.pone.0185647)
Supplement: S1 Appendix — (PDF) [file pone.0185647.s001.pdf]

S1 Appendix: Modifications to POSSUM to enable DW-MR simulation.

POSSUM is primarily designed for fMRI applications and so two modifications were made to enable the production of DW-MR datasets. Firstly, it only uses gradient-echo (GE) sequences, which mean the susceptibility-induced fields cause both geometric distortions and signal dropout in the data. Diffusion sequences use a spin-echo (SE), which means the susceptibility fields cause geometric distortions, but no dropout. In the first section we describe our implementation of SE sequences within POSSUM. Secondly, POSSUM does not produce images with diffusion weighting. In the second section, we describe how our SE version of POSSUM is incorporated into a recently proposed framework that enables POSSUM to produce diffusion-weighted data [1].

Implementation of spin-echo in POSSUM

Implementation of the spin-echo sequence in POSSUM was required in order to simulate DW-MR datasets with realistic susceptibility artefacts; i.e. geometric distortions but no signal dropout. SE sequences additionally enable the simulation of data with T_2 contrast. The implementation required several changes to POSSUM - an overview of how POSSUM works is shown in Fig 1 with all the changes made highlighted in red.

The most significant change to POSSUM is the modelling of the action of the spin-echo on the magnetisation. Typically, a spin-echo consists of an 180° RF pulse that rotates the magnetisation of each spin about a given axis, effectively reversing any phase accrued by the spin between excitation and the application of the pulse. In POSSUM, the smallest unit of magnetisation in an isochromat, a group of spins, and so it is necessary to model the action of the 180° pulse on an isochromat.

The 180° RF pulse affects both the magnitude and phase of the isochromat. Ordinarily, the isochromat's magnitude is reduced by the dephasing of its spins. The spin-echo reverses any loss of magnetisation that is caused by dephasing which is time-invariant, i.e. that from $T_2^{'}$ effects and susceptibility-induced gradients. The overall phase of the isochromat is altered by any magnetic fields that change the frequency of its precession: this is caused by both intended fields (e.g. the applied imaging gradients) and undesired fields (e.g. those induced by eddy-currents and susceptibility). The spin-echo reverses any phase accumulated by the isochromat between excitation and the 180° RF, leading to the cancellation of any additional phase accrual caused by undesired, time-invariant fields at the echo time.

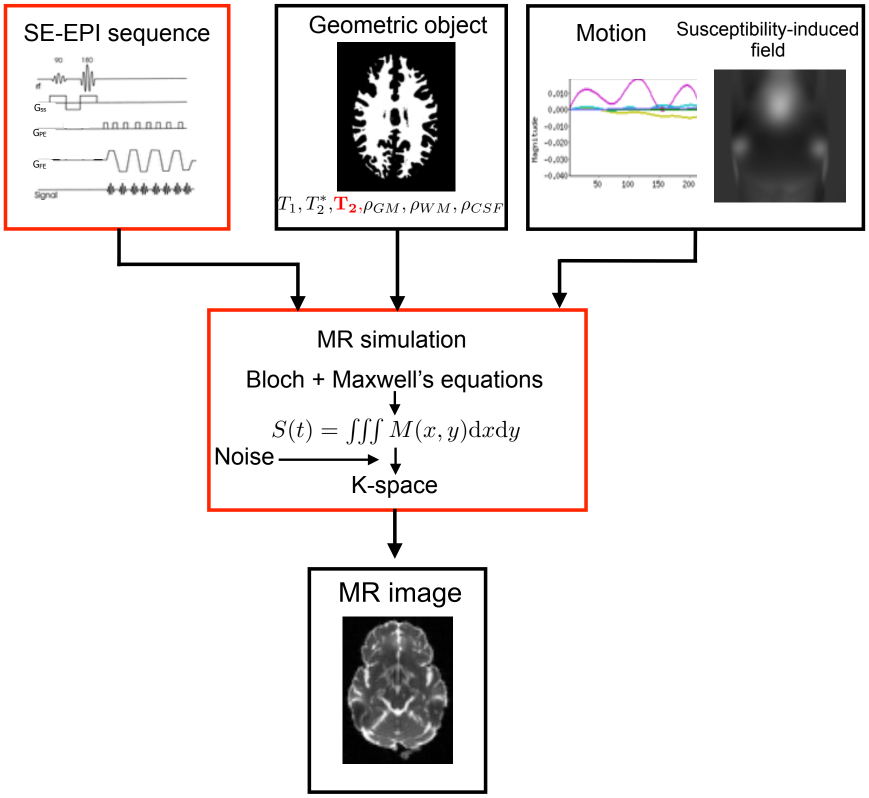

Figure 1: **Flowchart describing the POSSUM simulator.** Changes made in order to implement spin-echo in red. These changes are: addition of a 180 pulse to the input pulse sequence, inclusion of T2 tissue values as an input with the geometric object and modelling of the effects of the 180 pulse on the magnetisation in the main MR simulation phase.

The effect of the spin-echo on the isochromat's magnetisation was implemented using an approach similar to [2]. The main changes to the longitudinal magnetisation during an MR experiment are induced by T_2^* and T_2 effects. In the original POSSUM, T_2^* values were supplied for each tissue type, and the magnetisation was calculated according to:

$$m(t) = m(0) \exp\left(-\frac{t}{T_2^*}\right) \tag{1}$$

where t is the time since excitation of the isochromat. Using the relation $1/T_2^* =$

$1/T_2 + 1/T_2^{'}$, this may be rewritten:

$$m(t) = m(0)\exp\left(-\frac{t}{T_2}\right)\exp\left(-\frac{t}{T_2^{'}}\right) \tag{2}$$

To handle the effects of the spin-echo on $T_2^{'}$ induced magnetisation loss the magnetisation calculation becomes:

$$m(t) = m(0)\exp\left(-\frac{t}{T_2}\right)\exp\left(-\frac{|t_2 - RF_{dist}|}{T_2^{'}}\right) \tag{3}$$

where t_2 is the time since the last RF pulse and RF_{dist} is the time between the 90 and 180 pulses (0 if no spin-echo pulse has occurred). This can no longer be evaluated using a known T_2^* and the relationship $1/T_2^* = 1/T_2 + 1/T_2^{'}$; now both T_2^* and T_2 must be supplied to POSSUM, enabling it to calculate $T_2^{'}$. This formulation in Equation 3 is such that we observe rephasing of $T_2^{'}$ effects after a spin-echo pulse, and for a gradient echo sequence, $t_2 - RF_{dist} = t$ and we observe the expected T_2^* decay. The second factor leading to loss of longitudinal magnetisation is gradient-induced dephasing across the voxel. POSSUM handles this by evaluating an analytical function of the time-integral of these gradients [3]. The effects of the spin-echo here are modelled by reversing these integrals at the 180 RF pulse.

The effects of the spin-echo on the isochromat's phase are accounted for straightforwardly, by reversing any phase accumulated when the 180° RF occurs. A comparison of GE and SE images simulated in POSSUM is shown in Fig 2.

Incorporation of POSSUM into a DW-MR framework

In order to produce diffusion weighted datasets, the SE-enabled version of POSSUM was incorporated into a recently proposed framework that enables POSSUM to be used for the simulation of DW-MR datasets, with artefacts [1]. In brief, the framework creates diffusion-weighted geometric objects for use as input to POSSUM, producing DW-MR datasets as output. The framework additionally provides a displacement field, describing the geometric mapping of data distorted by artefacts (e.g. motion, eddy-currents and susceptibility) to a ground-truth space. An overview of the framework is presented in Fig 1 of the main article.

There are two main differences to the SE enabled framework, compared to that presented in [1]. Previously, the T_2 contrast was produced by using a GE-EPI sequence and replacing T_2^* values with T_2 values. In this implementation, T_2 contrast is simulated more faithfully through the use of a SE-EPI sequence. Furthermore, susceptibility artefacts can now be simulated realistically and are included in the framework. This means the output displacement fields now account for the geometric distortions caused by susceptibility-induced off-resonance fields. These are calculated according to [4]:

$$\boldsymbol{\psi}(\mathbf{r}) = t_s N f(\mathbf{r})\hat{\mathbf{p}} \tag{4}$$

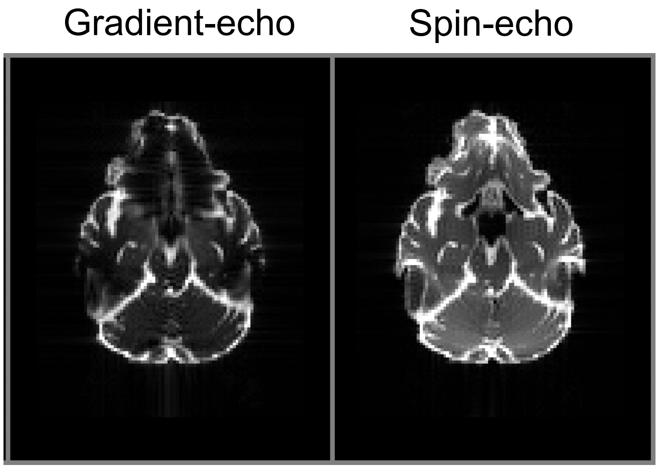

Figure 2: **Comparison between GE-EPI and SE-EPI images simulated in POSSUM.** Both images are displayed with the same maximum intensity. All acquisition parameters matched. Compared to the SE image, the GE image has less signal (due to T_2^* decay) and dropout in regions of large susceptibility induced-fields.

where $\boldsymbol{\psi}(\mathbf{r})$ is the field of spatial displacements in each DWI that result from susceptibility-induced off-resonance fields, defined at each location in the image $\mathbf{r}$, t_s is the echo spacing, N is the number of phase-encode lines, $f(\mathbf{r})$ is the susceptibility-induced off-resonance field and $\hat{\mathbf{p}}$ is a unit-vector that points along the phase-encode direction. The term $t_s N$ is also known as the readout time, and is the reciprocal of the bandwidth per pixel in the PE direction. The bandwidth per pixel is equal to the number of Hz in the off-resonance field that leads to a one-voxel displacement of signal along the PE direction.

References

[1] Graham MS, Drobnjak I, Zhang H. Realistic simulation of artefacts in diffusion MRI for validating post-processing correction techniques. NeuroImage. 2016;125:1079–1094. doi:10.1016/j.neuroimage.2015.11.006.

[2] Benoit-Cattin H, Collewet G, Belaroussi B, Saint-Jalmes H, Odet C. The SIMRI project: A versatile and interactive MRI simulator. Journal of Magnetic Resonance. 2005;173(1):97–115. doi:10.1016/j.jmr.2004.09.027.

[3] Drobnjak I, Gavaghan D, Süli E, Pitt-Francis J, Jenkinson M. Development of a functional magnetic resonance imaging simulator for model-

ing realistic rigid-body motion artifacts. Magnetic Resonance in Medicine. 2006;56(2):364–380. doi:10.1002/mrm.20939.

[4] Zaitsev M, Hennig J, Speck O. Point spread function mapping with parallel imaging techniques and high acceleration factors: Fast, robust, and flexible method for echo-planar imaging distortion correction. Magnetic Resonance in Medicine. 2004;52(5):1156–1166. doi:10.1002/mrm.20261.
