# Supplementary material for: Quantitative assessment of the susceptibility artefact and its interaction with motion in diffusion MRI
Source: PLoS One. 2017 Oct 2;12(10):e0185647. doi: 10.1371/journal.pone.0185647 (PMC5624609; doi:10.1371/journal.pone.0185647)
Supplement: S1 Table — As in Table 4, errors for FA, MD and the principle diffusion direction V1, but here divided into regions of interest based on the amount of distortion in the data. Values shown are the mean across the five noise realisations. V1 errors were only calculated in voxels with a ground-truth FA >0.2. Errors (calculated as the standard deviation of the mean value for each noise realisation) not shown as they were all 0 to 3 decimal places. (PDF) [file pone.0185647.s002.pdf]

		GT + noise	RB	FMB	MPB	MPB/F
SNR ∞	FA	0.000	0.049	0.024	0.019	0.016
	MD / $10^{-3}\,\mathrm{mm}^2\,\mathrm{s}^{-1}$	0.000	0.077	0.073	0.048	0.040
	V1 / degrees	0.000	3.602	1.693	1.008	0.687
SNR 40	FA	0.015	0.054	0.031	0.026	0.020
	MD / $10^{-3}\,\mathrm{mm}^2\,\mathrm{s}^{-1}$	0.015	0.154	0.110	0.078	0.072
	V1 / degrees	4.185	20.363	5.495	5.201	3.564
SNR 20	FA	0.049	0.083	0.054	0.052	0.036
	MD / $10^{-3}\,\mathrm{mm}^2\,\mathrm{s}^{-1}$	0.056	0.193	0.129	0.103	0.087
	V1 / degrees	12.470	21.283	11.352	11.865	9.057

(a) **Large distortion.** Errors in diffusion metrics averaged across all brain voxels with >6 mm geometric distortion.

		GT + noise	RB	FMB	MPB	MPB/F
SNR ∞	FA	0.000	0.021	0.016	0.012	0.010
	MD / $10^{-3}\,\mathrm{mm}^2\,\mathrm{s}^{-1}$	0.000	0.081	0.085	0.053	0.044
	V1 / degrees	0.000	4.923	1.919	1.276	0.826
SNR 40	FA	0.016	0.029	0.023	0.020	0.015
	MD / $10^{-3}\,\mathrm{mm}^2\,\mathrm{s}^{-1}$	0.021	0.092	0.105	0.059	0.049
	V1 / degrees	3.892	7.783	3.918	3.970	2.833
SNR 20	FA	0.050	0.052	0.045	0.047	0.033
	MD / $10^{-3}\,\mathrm{mm}^2\,\mathrm{s}^{-1}$	0.072	0.128	0.130	0.093	0.075
	V1 / degrees	11.736	12.663	9.858	10.976	7.875

(b) **Medium distortion.** Errors in diffusion metrics averaged across all brain voxels with >2 mm and <6 mm geometric distortion.

		GT + noise	RB	FMB	MPB	MPB/F
SNR ∞	FA	0.000	0.013	0.013	0.009	0.007
	MD / $10^{-3}\,\mathrm{mm}^2\,\mathrm{s}^{-1}$	0.000	0.070	0.066	0.043	0.036
	V1 / degrees	0.000	2.299	1.470	0.762	0.558
SNR 40	FA	0.016	0.023	0.019	0.019	0.013
	MD / $10^{-3}\,\mathrm{mm}^2\,\mathrm{s}^{-1}$	0.023	0.081	0.082	0.052	0.043
	V1 / degrees	3.711	4.836	3.589	3.665	2.586
SNR 20	FA	0.052	0.050	0.043	0.049	0.033
	MD / $10^{-3}\,\mathrm{mm}^2\,\mathrm{s}^{-1}$	0.083	0.132	0.118	0.097	0.078
	V1 / degrees	11.448	10.716	9.777	10.846	7.593

(c) **Small distortion.** Errors in diffusion metrics averaged across all brain voxels with <2 mm geometric distortion.

Table 1: As in Table 1C, errors for FA, MD and the principle diffusion direction V1, but here divided into regions of interest based on the amount of distortion in the data. Values shown are the mean across the five noise realisations. V1 errors were only calculated in voxels with a ground-truth FA >0.2. Errors (calculated as the standard deviation of the mean value for each noise realisation) not shown as they were all 0 to 3 decimal places.
